# The three-dimensional genome organization of *Drosophila melanogaster* through data integration

**DOI:** 10.1186/s13059-017-1264-5

**Published:** 2017-07-31

**Authors:** Qingjiao Li, Harianto Tjong, Xiao Li, Ke Gong, Xianghong Jasmine Zhou, Irene Chiolo, Frank Alber

**Affiliations:** 1 Molecular and Computational Biology, Department of Biological Sciences, University of Southern California, 1050 Childs Way, Los Angeles, CA 90089 USA; 2 Norris Comprehensive Cancer Center, Keck School of Medicine, University of Southern California, Los Angeles, CA 90089 USA; 3 Department of Pathology and Laboratory Medicine, David Geffen School of Medicine, University of California, Los Angeles, USA

**Keywords:** 3D genome structure, Higher order genome organization, Population-based modeling, Data integration, Hi-C, Lamina-DamID, Homologous pairing, *Drosophila melanogaster*, Heterochromatin

## Abstract

**Background:**

Genome structures are dynamic and non-randomly organized in the nucleus of higher eukaryotes. To maximize the accuracy and coverage of three-dimensional genome structural models, it is important to integrate all available sources of experimental information about a genome’s organization. It remains a major challenge to integrate such data from various complementary experimental methods. Here, we present an approach for data integration to determine a population of complete three-dimensional genome structures that are statistically consistent with data from both genome-wide chromosome conformation capture (Hi-C) and lamina-DamID experiments.

**Results:**

Our structures resolve the genome at the resolution of topological domains, and reproduce simultaneously both sets of experimental data. Importantly, this data deconvolution framework allows for structural heterogeneity between cells, and hence accounts for the expected plasticity of genome structures. As a case study we choose *Drosophila melanogaster* embryonic cells, for which both data types are available. Our three-dimensional genome structures have strong predictive power for structural features not directly visible in the initial data sets, and reproduce experimental hallmarks of the *D. melanogaster* genome organization from independent and our own imaging experiments. Also they reveal a number of new insights about genome organization and its functional relevance, including the preferred locations of heterochromatic satellites of different chromosomes, and observations about homologous pairing that cannot be directly observed in the original Hi-C or lamina-DamID data.

**Conclusions:**

Our approach allows systematic integration of Hi-C and lamina-DamID data for complete three-dimensional genome structure calculation, while also explicitly considering genome structural variability.

**Electronic supplementary material:**

The online version of this article (doi:10.1186/s13059-017-1264-5) contains supplementary material, which is available to authorized users.

## Background

It has become increasingly clear that a chromosome’s three-dimensional (3D) organization influences the regulation of gene expression and other genome functions. Early microscopy and biochemical studies showed that chromosomes in higher eukaryotes form distinct territories, which although stochastically organized tend to be located at preferred positions within the nucleus. For example, lamina-DamID experiments have identified specific chromatin domains with a high propensity to be located at the nuclear envelope (NE), confirming the important role of the NE in spatial genome organization and gene regulation in *Drosophila*, human, and mouse [1–3]. Chromosome conformation capture experiments (Hi-C and variants) detect chromatin interactions at a genome-wide scale [4–10] and reveal a hierarchical chromosome organization: the chromatin can be segmented into domains, which in turn combine to form subcompartments of functionally related chromatin [6, 11, 12]. Topological associated domains (TADs) are defined by observing an increased probability of interaction between chromatin regions in a domain relative to interactions between domains. In addition, it has been shown that the border regions between domains are enriched in specific insulator proteins, such as CTCF and ZNF143 in mammalian cells and BEAF, CTCF, and CP190 in *Drosophila* cells. However, the precision of domain border detection depends to some extent on the sequencing depth as well as algorithmic parameter settings. At increased sequencing depth it is possible to detect reliably individual chromatin loops, which often demarcate contact domains (at ~100 kb domain length) [9].

Computational approaches can aid in mapping the global 3D structures of genomes at various scales. These can be divided into data-driven and physics-based de novo simulation techniques [13]. Recent physics-based polymer models of chromosomal regions provided critical insights into mechanisms of chromatin loop formation, such as the “loop extrusion” (LE) models [14, 15] and “strings and binders switch” (SBS) models [16, 17] as well as related approaches [18–20]. Data-driven approaches use experimental information, often Hi-C data, to generate 3D genome structures that are constrained to be consistent with the data. These approaches can be divided into three classes [21, 22]. The first represents the genome as a consensus structure [23–30]. Contact frequencies are usually transformed to spatial distances assuming an anti-correlation between the two metrics [23, 24, 28, 31, 32]. The distance matrix is then used to generate a consensus model by optimizing a scoring function, by Bayesian interference or generalized linear models. By definition, consensus models cannot reflect the considerable structural variability of genomes between individual cells. To incorporate aspects of structural variability, resampling methods (such as TADbit [21, 32, 33]) perform many independent optimizations of the same scoring function to resample an ensemble of structures from random starting configurations [31–35]. Other resampling methods use chromatin contact restraints, which omit the need to relate contact frequencies to spatial distances [36–39]. Chrom3D is such a method that uses the most significant chromatin contacts from Hi-C experiments and restrains lamina associated chromatin domains (LADs) to the NE (from lamina-DamID experiments) [39]. Common to all resampling methods is that the input dataset is applied to each individual structure, and often the restraint violations due to conflicting data lead to structural variance in the resampled ensemble.

Conceptually different from these methods are population-based deconvolution (PD) approaches. These methods attempt to de-convolve ensemble Hi-C data into a large population of structures, so that the accumulated chromatin contacts of all structures reproduce the ensemble Hi-C data rather than attempting to impose the data on each structure [5, 40–42]. PD methods allow structures to be in different conformational states that can contain contacts that would otherwise be mutually exclusive when imposed on all structures. As a result, they can reproduce well almost all the chromatin contacts from Hi-C experiments and avoid unphysical structures from simultaneous enforcement of conflicting restraints. These methods generate structures that can almost entirely satisfy all the contact restraints without restraint violations. Also, these approaches do not need to assume any functional relationship between contact frequencies and spatial distances. We previously introduced one of the first Hi-C-based PD methods for modeling complete diploid genomes (PGS, population-based genome modeling [5, 22, 42]) and extended its applicability to various human and mouse cells [43]. We use an iterative, probabilistic optimization framework to deconvolve the Hi-C data into a population of individual structures by inferring cooperative chromatin interactions that are likely to co-occur in the same cells [22, 42]. Our method generates a large number of genome structures whose chromatin contacts are statistically consistent with those from the Hi-C data. These structures describe interactions between TADs for the whole diploid genome. Other PM approaches generate chromosome structures by sampling from a molecular dynamics trajectory. For example, a maximum entropy method was used to reproduce experimental Hi-C maps of individual chromosomes [40]. In other work, chromatin was divided into a few functional states and the only parameters were the chromatin state binding affinities [44]. In an earlier method a polymer model combined with Monte Carlo sampling was used to study chromatin conformations within TADs from ensemble 5C data [41].

So far, most PD models of genome structures have typically relied on just one data type, such as Hi-C, even though a single experimental method cannot capture all aspects of the spatial genome organization. However, data are available from a wide range of technologies with complementary strengths and limitations. Integrating all these different data types would greatly increase the accuracy and coverage of genome structure models. Moreover, such models would offer a way to cross-validate the consistency of data obtained from complementary technologies. For example, lamina-DamID experiments show a chromatin region’s probability to be close to the lamina at the nuclear envelope, while Hi-C experiments reveal the probability that two chromatin regions are in spatial proximity. Large-scale 3D fluorescence in situ hybridization (FISH) experiments show the distance between loci directly, and can be used to measure the distribution of distances across a population of cells.

It remains a major challenge to develop hybrid methods that can systematically integrate data from many different technologies to generate structural maps of the genome. In this paper, we present a method for integrating contact frequency information from Hi-C and lamina-DamID experiments to maximize the accuracy of population-based 3D genome structural models generated by data deconvolution. We apply this approach to model the diploid genome of *Drosophila*.

*Drosophila melanogaster* is a popular model organism to study the organization and functional relevance of 3D genome structure, owing to its relatively small genome and the availability of many genetic tools. A variety of microscopy-based experiments have already studied the nuclear organization of *D. melanogaster* and elucidated some regulatory mechanisms [45–49]. For example, the pairing of homologous chromosomes has been observed in the somatic cells of *D. melanogaster* and other dipteran insects [50–53]. This kind of pairing can influence gene expression by forming interactions between regulatory elements on homologous chromosomes, a process called transvection [48, 54]. Although transvection is common in *Drosophila*, not every gene region with homolog pairing responds to this regulation. Therefore, questions remain as to whether somatic homolog pairing has other regulatory roles. In *Drosophila*, the centromeres tend to cluster and are positioned at the periphery of the nucleolus during interphase [55]. Centromere clustering is also observed in many other organisms, including yeast, mouse, and human, and this process is thought to play an important role in determining the overall genome architecture [56, 57].

Over the past 10 years, high-throughput genetic and genomic techniques have generated genome-wide maps of histone modifications, transcription factor binding, and chromatin interactions for *D. melanogaster* [1, 7, 8, 58, 59]*.* Pickersgill et al. [1] used lamina-DamID experiments combined with a microarray technique to detect the binding signals of genome-wide chromatin to the lamina matrix in *Drosophila* Kc embryonic cells. Around 500 genes were detected to interact with the lamina. These genes were transcriptionally silenced and late-replicating. Pickersgill et al. then used FISH experiments to confirm that the lamina-targeted loci were more frequently located at the nuclear envelope than other loci. Recently, genome-wide chromatin contacts have also been determined for 16–18-h *Drosophila* embryos using the Hi-C technique [8]. The euchromatin genome (excluding pericentromeric heterochromatin) was divided into 1169 physical domains based on Hi-C interaction profiles. These physical domains (which would be referred to as TADs in mammalian cells) were assigned to four functional classes based on their epigenetic signatures: null, active, Polycomb-group (PcG), and HP1/centromere.

Despite all this work, the global 3D nuclear architecture of the *D. melanogaster* genome is still unknown. Because both Hi-C and lamina-DamID data are available for *Drosophila* embryonic cells, we used these data to test our integration method. Each diploid genome structure in our population-based model is defined by the 3D positions of all 1169 TADs. The structures are generated by optimizing a likelihood function, so that the ensemble is statistically consistent with both the experimentally derived contact probabilities between all chromatin domains from Hi-C data and the probability that a given chromatin domain is close to the NE from lamina-DamID data.

We validated our 3D genome models against independent experimental data and known structural features. Our models confirm the formation of distinct chromosome territories, with relatively low rates of intermingling between chromosomes [60, 61]. In addition, our models often show a polarized organization of chromosomes in the nucleus [45, 62, 63]. Analysis of the model population leads also to a number of new insights about the nuclear organization of *D. melanogaster* and its functional relevance. For instance, our models reveal the preferred locations of heterochromatin and the nucleolus, which we were able to confirm by 3D FISH and immunofluorescence experiments. The nucleolus serves as an anchor for chromosomes and is surrounded by pericentromeric heterochromatin. The distance of pericentromeric heterochromatin regions from the periphery varies by chromosome, with chromosome 4 and X heterochomatin more peripheral relative to pericentromeric regions of other chromosomes. Interestingly, the frequency of homologous pairing varies along the chromosomes, with the lowest frequencies observed in our models for domains enriched in protein binding sites for Mrg15. These observations support the model that Mrg15 plays a role in the dissociation of homologous chromosome pairs during interphase, as previously suggested [64]. Finally, the structure population suggests that homologous chromosome pairing plays a functional role in transcriptional activity and the DNA replication program.

## Results

### Population-based genome structure modeling from data integration

Our goal is to determine a population of 3D genome structures for *D. melanogaster* that is consistent with data from Hi-C and lamina-DamID experiments. Suppose **A** is a probability matrix derived from Hi-C data, and *E* is a probability vector derived from lamina-DamID data. The elements of **A** describe how frequently a given pair of TADs are in contact with each other in an ensemble of cells, and *E* describes how frequently a given TAD is in contact with the nuclear envelope (NE). The goal is to generate a population of genome structures **X**, whose TAD–TAD and TAD–NE contact frequencies are statistically consistent with both **A** and *E*. We formulate the genome structure modeling problem as a maximization of the likelihood *P*(**A**, *E*|**X**).

More specifically, the structure population is defined as a set of *M* diploid genome structures **X** = {**X**_1_, **X**_2_, …, **X**_*M*_}, where the *m*-th structure **X**_*m*_ is a set of 3D vectors representing the center coordinates of 2 *N* domain spheres ${\mathbf{X}}_m=\left\{{\overrightarrow{x}}_{im}:{\overrightarrow{x}}_{im}\in {\Re}^3,\kern1em i=1,\kern0.5em 2\dots, 2N\right\}$. *N* is the number of TADs, but each domain has two homologous copies with different coordinates. The contact probability matrix **A** = (*a*_*IJ*_)_*N* × *N*_ for *N* domains is derived from the Hi-C data, which do not distinguish between homologous copies (see “Methods”; Additional file 1: Supplementary methods A.2). Each element *a*_*IJ*_ is the probability that a direct contact between domains *I* and *J* exists in a structure of the population. (Note that the capital letter indices *I* and *J* refer to domains without distinguishing between their homologous copies, while the lowercase indices *i*, *i’* and *j*, *j’* do distinguish between copies). The contact probability vector *E* = {*e*_*I*_|*I* = 1, 2, …, *N*} is derived from the lamina-DamID data and defines the probability for each TAD to be localized at the NE. With known **A** and *E*, we calculate the structure population **X** such that the likelihood *P*(**A**, *E*|**X**) is maximized.

The Hi-C and lamina-DamID experiments provide data that are averaged over a large population of cells, so they cannot reveal which contacts co-exist in the same 3D structure. Therefore, both **A** and *E* are interpreted as ensemble averages. To represent information derived from individual cells, we introduce two latent variables, **W** and **V**. The “contact indicator tensor” **W** = (*w*_*ijm*_)_2*N* × 2*N* × *M*_ is a binary, third-order tensor. It contains the information missing from the Hi-C data **A**, namely which domain contacts belong to each of the *M* structures in the model population and also which homologous chromosome copies are involved (*w*_*ijm*_ = 1 indicates a contact between domain spheres *i* and *j* in structure *m*; *w*_*ijm*_ = 0 otherwise). **W** is a detailed expansion of **A** into a diploid, single-structure representation of the data. The structure population **X** is consistent with **W**. Therefore, the dependence relationship between these three variables is given as **X** → **W** → **A**. Another latent variable, **V** = (*v*_*im*_)_2*N* × *M*_, specifies which domain is located near the NE in each structure of the population and also distinguishes between the two homologous TAD copies (*v*_*im*_ = 1 indicates that TAD *i* is located near the NE in structure *m*; *v*_*im*_ = 0 otherwise). The dependence relationship between **X**, **V**, and *E* is given as **X** → **V** → *E*, because **X** is the structure population consistent with **V** and **V** is a detailed expansion of *E* at a diploid and single-structure representation of the data.

In addition to the Hi-C and lamina-DamID data, we also consider additional information specific for *Drosophila* genome organization, e.g., the nuclear volume, an upper bound for homolog chromosome pairing, constraints connecting consecutive domains (including heterochromatin domains), as well as constraints for anchoring centromeres to the nucleolus (see the detailed description in the “Methods” section).

Thus, the optimization problem is expressed as:

1 $$ {\displaystyle \begin{array}{l}\widehat{\mathbf{X}}=\arg \kern0.1em \underset{\mathbf{X},\mathbf{W},\mathbf{V}}{\max \limits}\kern0.5em \log \kern0.5em P\left(\mathbf{A},E,\mathbf{W},\mathbf{V}\left|\mathbf{X}\right.\right)\hfill \\ {}\mathrm{s}\mathrm{ubject}\  \mathrm{to}\left\{\begin{array}{l}\mathrm{s}\mathrm{patial}\  \mathrm{constraint}\ \mathrm{I}:\mathrm{nuclear}\  \mathrm{volume}\  \mathrm{constraint}\mathrm{s}\hfill \\ {}\mathrm{s}\mathrm{patial}\  \mathrm{constraint}\ \mathrm{I}\mathrm{I}:\mathrm{excluded}\  \mathrm{volume}\  \mathrm{constraint}\mathrm{s}\hfill \\ {}\mathrm{s}\mathrm{patial}\  \mathrm{constraint}\ \mathrm{I}\mathrm{I}\mathrm{I}:\mathrm{chromosome}\  \mathrm{pairing}\  \mathrm{upper}\  \mathrm{bound}\hfill \\ {}\mathrm{s}\mathrm{patial}\  \mathrm{constraint}\ \mathrm{I}\mathrm{V}:\mathrm{consecutive}\  \mathrm{domain}\  \mathrm{constraint}\hfill \end{array}\right.\hfill \end{array}} $$

The log likelihood can be expanded as

2 $$ {\displaystyle \begin{array}{ll}\log P\left(\mathbf{A},E,\mathbf{W},\mathbf{V}|\mathbf{X}\right)\hfill & =\log P\left(\mathbf{A},E|\mathbf{W},\mathbf{V}\right)P\left(\mathbf{W},\mathbf{V}|\mathbf{X}\right)\hfill \\ {}\hfill & =\log P\left(\mathbf{A}|\mathbf{W}\right)P\left(E|\mathbf{V}\right)P\left(\mathbf{W},\mathbf{V}|\mathbf{X}\right)\hfill \end{array}} $$

**Fig. 1:**
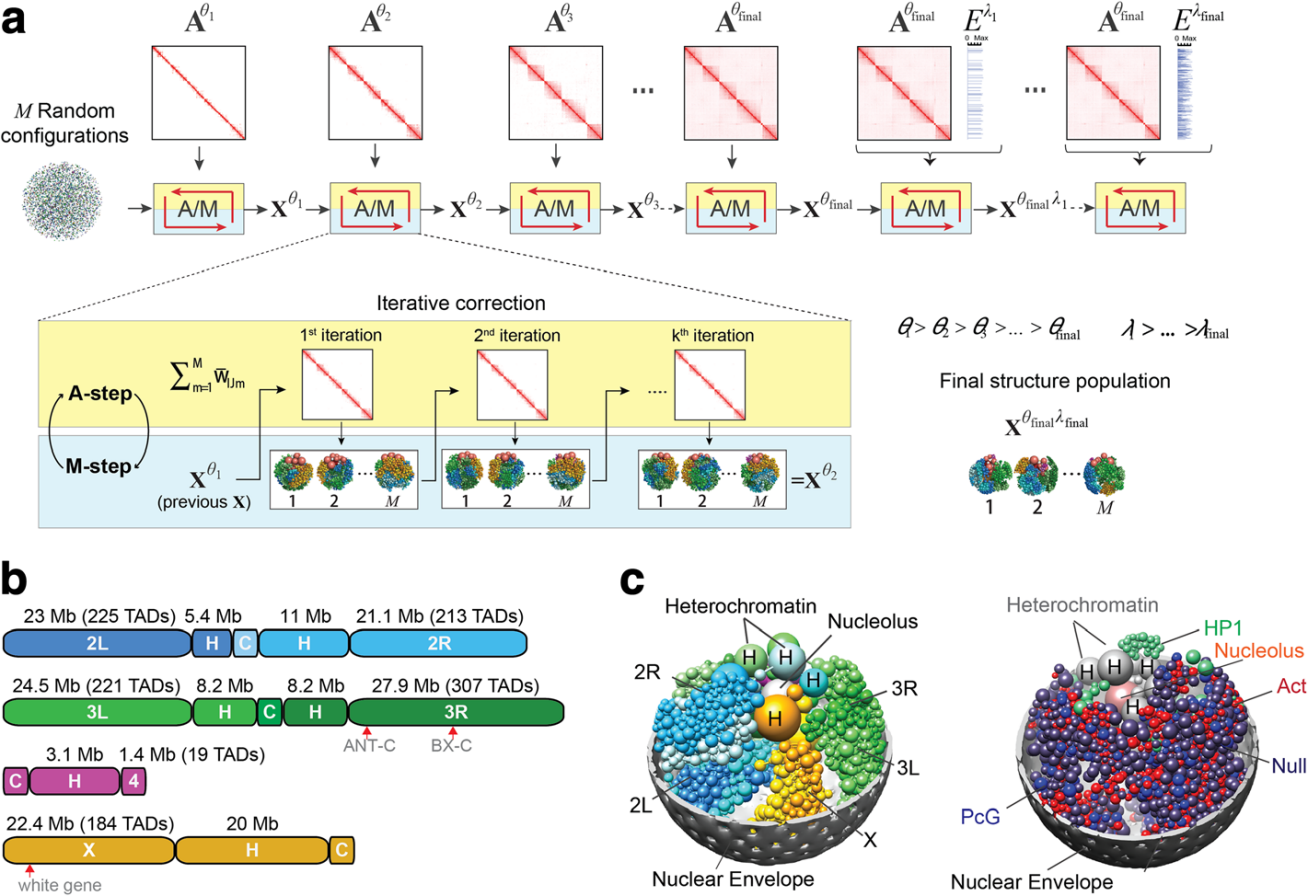
Overview of the population-based genome structure modeling approach and its application to the *Drosophila* genome. **a** The initial structures are random configurations. Maximum likelihood optimization is achieved through an iterative process with two steps, assignment (*A*) and modeling (*M*). We increase the optimization hardness over several stages by including contacts from the Hi-C matrix **A** with lower probability thresholds (*θ*). After the population reproduces the complete Hi-C data, we include the vector *E* (lamina-DamID), again in stages with decreasing contact probability thresholds (*λ*). **b** Schematic of the *Drosophila* genome. The autosome arms are designated 2L, 2R, 3L, 3R, 4, and X. The arms of chr2 and chr3 are connected by centromeres labeled “*C*”. Euchromatic regions are labeled as the arm. The *numbers* along the *top* of a genome indicate the length of the section in megabases (Mb), and for euchromatin the number of spheres (*TADs*) in the structure model is also given. The heterochromatic region of each chromosome arm is labeled “*H*”. The *white gene* is located ~19 M away from the heterochromatin of chrX. Also indicated are the Hox genes: five genes of the Antennapedia complex (*ANT-C*) are located at ~2.3–2.8 Mb from the heterochromatin of chr3R, and three genes of the Bithorax complex (*BX-C*) are located at ~12.4–12.7 Mb from the heterochromatin of chr3R. **c** Snapshot of a single structure randomly picked from the final population. *Left panel*: The full diploid chromosomes are shown in colors: *blue*, chr2; *green*, chr3; *magenta*, chr4; *orange*, chrX. The two homologs of the same chromosome are distinguished by the color tone, with one homolog copy with lighter and one with darker color. The heterochromatin spheres are larger than the euchromatin domains. The nucleolus is colored in *silver. Right panel*: The euchromatin domains are colored to reflect their epigenetic class: *red*, active; *blue*, PcG; *green*, HP1; *dark purple*, null. Heterochromatin spheres are shown in *grey* and the nucleolus in *pink*

We have developed a variant of the EM method to iteratively optimize the log likelihood [42]. Each iteration consists of two steps (Fig. 1a):

- Assignment step (*A-step*): Given the current model **X** ^(*i*)^, estimate the latent variables **W** ^(*i* + 1)^ and **V** ^(*i* + 1)^ by maximizing the log-likelihood over all possible values of **W** and **V**:
  3 $$ {\mathbf{W}}^{\left(i+1\right)},{\mathbf{V}}^{\left(i+1\right)}=\arg\ \underset{\mathbf{W},\mathbf{V}}{\max}\kern0.5em \log \kern0.5em P\kern0.5em \left(\mathbf{A}\left|\mathbf{W}\right.\right)P\left(E\left|\mathbf{V}\right.\right)P\left(\mathbf{W},\mathbf{V}\left|{\mathbf{X}}^{(i)}\right.\right) $$
- Modeling step (*M-step*): Given the current estimated latent variables **W** ^(*i* + 1)^ and **V** ^(*i* + 1)^, find the model **X** ^(*i* + 1)^ that maximizes the log-likelihood function:
  4 $$ {\mathbf{X}}^{\left(i+1\right)}=\arg \kern0.5em \underset{\mathbf{X}}{\max}\kern0.5em \log \kern0.5em P\left(\mathbf{A}{\left|\mathbf{W}\right.}^{\left(i+1\right)}\right)P\left(E{\left|\mathbf{V}\right.}^{\left(i+1\right)}\right)P\left({\mathbf{W}}^{\left(i+1\right)},{\mathbf{V}}^{\left(i+1\right)}\left|\mathbf{X}\right.\right) $$

The detailed implementation of the A-step and M-step is described in “Methods”. We follow the step-wise optimization strategy described previously [42] and gradually increase the optimization hardness by adding contact constraints at a decreasing contact probability threshold.

### A population of *Drosophila* genome structures at the TAD level

The euchromatin regions of *D. melanogaster* chromosomes 2, 3, 4, and X are partitioned into 1169 TADs, as previously described [8]. The region of pericentromeric heterochromatin of each chromosome arm is spatially clustered and represented by a single domain (Fig. 1b; “Methods”) [65–67]. The nuclear diameter is set to 4 microns. The model also contains a nucleolus, represented by a sphere with a radius one-sixth of the nuclear radius. We estimated the nucleolus volume from our immunofluorescence analysis of *Drosophila* Kc cells (Additional file 1: Figure S7a).

By optimizing the likelihood function (Eq. 1) we generated a population of 10,000 genome structures that accurately reproduce the domain contact probabilities from Hi-C experiments and the probabilities for domains to reside at the NE from lamina-DamID experiments (“Methods”). Our approach produces structures that almost entirely satisfy all the imposed contact restraints without restraint violations: 99.999% of all imposed contact restraints are satisfied (at a tolerance of 0.05; Additional file 1: Figure S13), showcasing the excellent agreement of all the contacts derived from the Hi-C map and the structure population. For comparison, we also generated a population of structures using only Hi-C data, referred to hereafter as a control model. To test the reproducibility of our method, we also generated a replicated, independently calculated structure population by rerunning our modeling pipeline with the same parameters but different random staring configurations of all domains. The replicated structure population confirms our conclusions (Additional file 1: Figure S10).

### Validation of the structure population

#### Reproducing the Hi-C contact probabilities

**Fig. 2:**
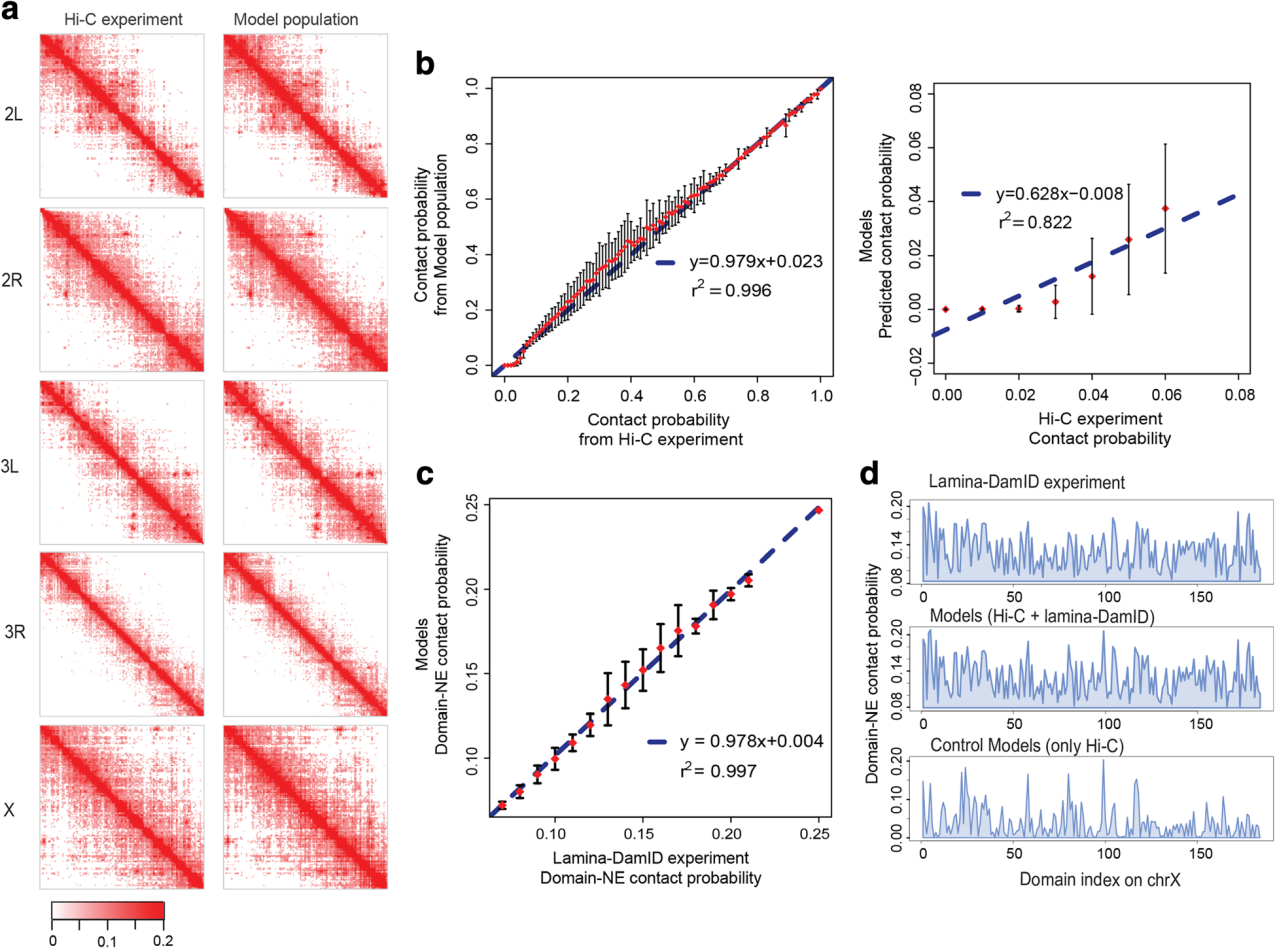
Reproduction of Hi-C and lamina-DamID data. **a** Heat maps of intra-arm contact probabilities from Hi-C experiments (*left*) and intra-arm contact frequencies from the structure population (*right*). Their similarity is quantified by element-wise Pearson’s correlations, which are 0.984, 0.985, 0.984, 0.986, and 0.980 for chr2L, chr2R, chr3L, chr3R, and chrX, respectively. The maps only show interactions with probabilities no less than 6%, which are used as constraints in our modeling procedure. We set the darkest color for probability = 0.2 and above to avoid making regions away from the diagonal (long range interactions) too weak and blank for comparison. **b** Agreement between the experimental data and model contact probabilities. *Left panel*: The input Hi-C contact probabilities are divided into 100 bins, the corresponding model contact probabilities in one bin are summarized by mean and variance, and then the error bar plot is shown. The *blue dashed line* is the linear regression line between the average model contact probabilities of each bin and the mid-point Hi-C contact probabilities of the bins. Their Pearson’s correlation is 0.998 with *p* value <2.2e − 16. *Right panel*: Close-up of the agreement between experiment and model for contacts with probabilities less than 6%, which are not used as constraints in our modeling procedure. In this range, the Pearson’s correlation is 0.907 with *p* value = 4.87e − 3. **c** The agreement between NE association frequencies from lamina-DamID experiments and the model population. This figure is plotted in the same way as **b**. The structure population well reproduces the input frequencies derived from lamina-DamID data, with a Pearson’s correlation of 0.95 and *p* value <2.2e − 16. **d** Comparison of experimental and model lamina-DamID frequencies on chrX. The *top panel* shows the input frequencies derived from the lamina-DamID signal, the *middle panel* shows the fraction of domains located at the NE in the structure population obtained by Hi-C and lamina-DamID data integration, and the *bottom panel* shows the fractions obtained in our control structure population generated using only Hi-C data

We first assessed the consistency between the chromatin contact probabilities in our structure population and those observed experimentally. The contact probability of any two domains is defined as the fraction of model genome structures for which the two domains are in physical contact with each other, measured over the entire population (a domain–domain contact is defined by an overlap between the domains’ soft sphere contact radii). The domain contact probability matrix in our model shows excellent agreement (high correlation) with the Hi-C data, and also closely reproduces the interaction patterns visible in the matrix. The average column-based Pearson’s correlation coefficient (PCC) is 0.984, and the element-wise PCC is 0.984 (Additional file 2: Table S1). The correlation coefficients of the intra-chromosome arm contact probabilities range between 0.980 and 0.998 over all arms, confirming the excellent visual comparison shown in Fig. 2a. The correlation coefficients for inter-arm and inter-chromosome contact probabilities are lower, ranging between 0.148 and 0.382 (Additional file 2: Table S1). This relatively weak agreement between the model and the experimental data for inter-arm and inter-chromosome interactions can be explained by the following argument. In the Hi-C data, inter-arm and inter-chromosome interactions are relatively infrequent and unstructured, indicating that contacts between chromosomes are predominantly random. Due to their low occurrence, these interactions are also less reproducible than intra-arm interactions, especially at low sequencing depth. This reasoning is confirmed by comparing two Hi-C experiments performed with two different restriction enzymes [5, 68]. The differences in contact frequencies between the two experiments are generally much larger for inter-chromosome arm interactions than for intra-chromosome arm interactions. We do not use Euclidian distance to measure the similarity between the domain contact probability from the model and Hi-C experiment for two reasons: first, the intra-chromosome arm contacts are much higher than inter-chromosome/inter-arm contacts, which make the values not comparable between the groups; and second, there is no standard value to determine how small the distance is to indicate a good agreement.

Another quality measure for our models is how well we can predict the frequencies of chromatin interactions that were not included as constraints in the optimization. In our models, we did not impose constraints for any pair of TADs whose contact probability was lower than *a*_*ij*_ = 0.06. Very low contact probabilities are expected to contain a higher fraction of experimental noise. Such pairs include ~99.99% of all inter-chromosome and inter-chromosome arm interactions. However, our structure population is capable of predicting the missing data (Fig. 2b, right panel). Many of the low-frequency contacts are formed as a consequence of imposing more significant interactions (with contact probabilities *a*_*ij*_ ≥ 0.06), and their correct prediction is a good indicator of the model quality.

#### Reproducing the lamina-DamID binding frequency

Lamina-DamID experiments identify the probability that a locus is associated with the NE (more precisely, with the lamina protein located at the NE). We first assess the consistency between our structure population and the lamina-DamID experiment (a TAD–NE contact is defined when the domain surface is less than 50 nm from the NE). The association probabilities are in excellent agreement, with a Pearson’s correlation of 0.95 (Fig. 2c, d; Additional file 1: Figure S1a). Recalling that the TADs of *Drosophila* are divided into four functional classes, we find that TADs in the active class are less frequently in contact with the NE than those from the other three classes (HP1, PcG, and null; Additional file 1: Figure S1a). This result agrees with prior observations in the literature that the genes interacting with lamina are usually transcriptionally silent and lack active histone marks [1]. The control population generated using only Hi-C data also shows good (albeit substantially lower) correlations between its NE association probabilities and the lamina-DamID experiments (Pearson’s correlation is 0.64, with *p* value <2.2e − 16) (Fig. 2d; Additional file 1: Figure S1b). This relatively high correlation value in the control population shows a strong consistency between the Hi-C-based models and the independent lamina-DamID data and confirms the generally good quality of our Hi-C-based structure modeling.

#### Agreement with FISH experiments

Our genome structures also predict well the NE association frequencies observed by independent FISH mapping of 11 different genomic loci [1]. The Spearman’s rank correlation coefficient between experiment and model is 0.642 for these loci, with a significant *p* value = 3.31e − 2 (Additional file 1: Figure S2a). Notably, the corresponding correlation with the control structure population (only using Hi-C data) is substantially lower (Spearman’s rank correlation coefficient = 0.38 with *p* value = 0.25) (Additional file 1: Figure S2b), demonstrating the benefit of data integration to generate more accurate genome structures.

#### Presence of chromosome arm territories

Chromosome territories have been observed directly in higher eukaryotes, including mammalian cells [69, 70]. In *Drosophila*, chromosome territories can be inferred from the fact that Hi-C contact frequencies between chromatin regions in the same chromosome arms are substantially higher than those between chromosome arms [7, 8]. Previous 4C experiments on larval brain tissue confirm the limited nature of interactions between genes on different chromosome arms [61]. FISH experiments have also suggested chromosome territories in *Drosophila* [60]. In our models, we analyze the formation of chromosome territories by calculating a territory index (TI), which measures the extent of chromosome mixing [71]. To calculate TI in each structure, first we define the spanning volume of each chromosome, which is the surface convex hull of all its domain positions [71]. TI is then defined as the percentage of all domains occupying the chromosome spanning volume of the target chromosome (Additional file 1: Supplementary methods C.2). By definition, the maximum TI value of 1 indicates that the chromosome’s spanning volume is exclusively occupied by its own domains, and therefore experiences limited chromosome mixing. When considering domains from homolog chromosome copies, the territorial index ranges between 0.96 and 1.0 for all the chromosome arms (Additional file 1: Figure S3a; Additional file 2: Table S2). When separating the homolog chromosomes, however, the TI values range between 0.62 and 1.0 for the larger chromosome arms (Additional file 1: Figure S3b), suggesting that homolog chromosome pairs share almost the same territory due to strong homolog pairing.

#### Residual polarized organization

**Fig. 3:**
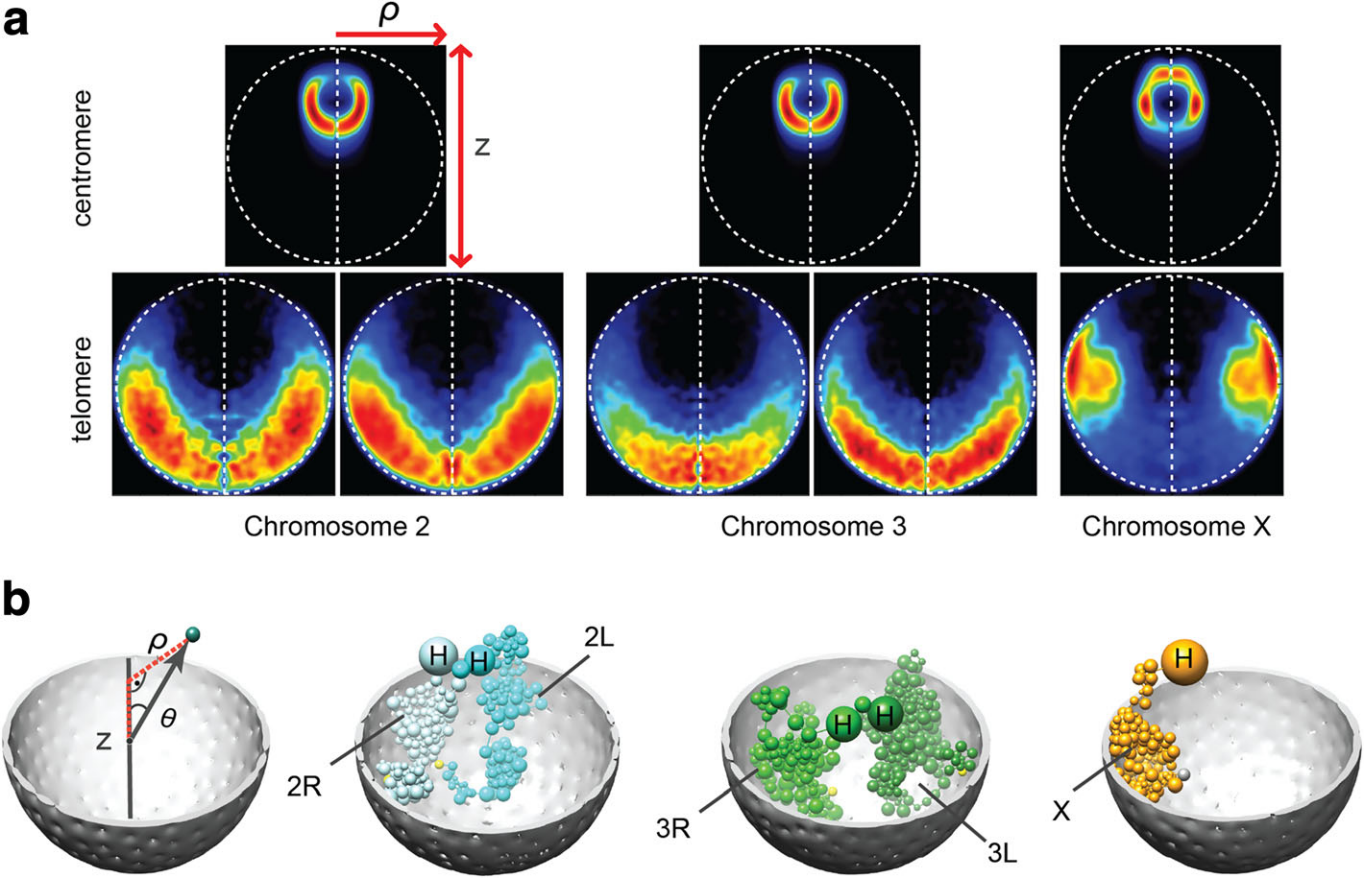
Residual polarized organization. **a** Projected localization probability densities (LPDs) of centromeres and peri-telomeric sequences for all chromosome arms calculated from the structure population. Probability densities are determined with respect to two principle axes of the nuclear architecture. The z-axis connects the center of the nucleolus with the origin at the nuclear center. The radial axis defines the distance of a point from the central z-axis (shown in the left panel in **b**). The left half of the projected localization density plot mirrors the right half for visual convenience. **b** The genome organization for different chromosome arms in one genome structure

In a polarized genome organization, each chromosome occupies an elongated territory with the centromere at one nuclear pole and telomeres on the opposite side of the nucleus. Such an organization, called Rabl, typically occurs after mitosis and has been observed in a variety of plants [72], yeast, and both polytene and non-polytene *Drosophila* nuclei; it is also common in *Drosophila* embryos [45, 62, 63]. In the majority of our genome structures (67.4%; Additional file 1: Supplementary methods C.3), more than half of the chromosomes arms (chr2L, chr2R, chr3L, chr3R, and chrX) are organized with their centromeres and telomeres located in opposite nuclear hemispheres (Additional file 1: Figure S4b–d). This organization is also apparent when calculating the localization probabilities of chromosomes, which are highest for the telomeres in a region near the NE opposite to their respective centromeres (Fig. 3a, b). Taken together, these results suggest that interphase chromosomes retain some features of Rabl organization.

#### Nuclear colocalization of Hox gene clusters

In *Drosophila*, the two PcG-regulated Hox gene clusters (Antennapedia complex and Bithorax complex) tend to co-localize in the head of 10–11-stage embryos [73], despite being separated by 10 Mb in sequence on chromosome 3 (Fig. 1b). To test their spatial colocalization in our models, we calculate the pairwise spatial distances between the two gene clusters in every structure of the population (Additional file 1: Supplementary methods C.4). As a random control, we also calculate the pairwise distances between 30 pairs of gene clusters that only contain repressive TADs and share similar chromatin features in order to mimic the PcG-regulated Hox genes. In this control group each pair of gene clusters contains the same number of repressive domains, and are separated by the same sequence distance, as the pair of Hox gene clusters (Additional file 1: Supplementary methods C.4). We define a colocalization if the closest surface-to-surface distance among the domain pairs between two clusters is less than 200 nm (Additional file 1: Figure S5a also shows results when varying this cutoff). In good agreement with the experiment, the Hox gene clusters are colocated in about 17.2% of our structure population, a substantially higher rate than that observed in the control groups (median value 7.2%). Only one pair of clusters among the 30 control groups is more frequently colocated than the Hox gene clusters (Additional file 1: Figure S5b). This gene cluster is brought together by nearby active domains, which form frequent interactions.

#### White *gene localizing near pericentromatic heterochromatin*

Position-effect variegation (PEV) is a process whereby a euchromatic gene is transcriptionally silenced through an abnormal juxtaposition with heterochromatin, due to chromosome rearrangements or transpositions. PEV has been intensively studied for the *Drosophila white* gene [74, 75], which is on the distal end of chromosome X and separated by more than 19 Mb from pericentromeric heterochromatin (Fig. 1b). A chromosome inversion can insert the *white* gene in sequence next to pericentromeric heterochromatin, which leads to its repression. Hence, such chromosomal rearrangement may be favored if the *white* gene has an increased chance of being in spatial proximity to the heterochromatin. However, technical limitations prevent us from directly measuring contacts between the white gene and heterochromatin with Hi-C experiments. Using our structure population, we can measure how often the *white* gene is located close to pericentromeric heterochromatin of chromosome X. As a control set, we took the four domains that are located at equivalent sequence distances to the pericentromeric heterochromatin on chromosomes 2 and 3.

Interestingly, the spatial distance between the *white* gene and the X chromosome heterochromatin is significantly smaller than the corresponding distances of the control groups (one-tailed Welch’s two sample t-test, *p* value <2.2e − 16) (Additional file 1: Figure S6a). Although it is unlikely for distal loci to come together in three dimensions, we found that in ~1.3% of structures the *white* gene and pericentromeric heterochromatin of chromosome X were justaposed (positioned within a distance of 200 nm; Additional file 1: Figure S6b). This frequency is nine times larger than the colocalization frequency in the control sets (0.14% of structures). Therefore, our models suggest that the *white* gene is more frequently located near pericentromeric heterochromatin compared to equivalent sites on other chromosomes. This result suggests that spatial proximity facilitates the occurrence of the *white* gene translocation next to pericentromeric heterochromatin in living cells.

### Different chromosome domains have distinct preferred locations in the nucleus

The evidence listed above demonstrates the consistency of our models with experimental data and known properties of the *Drosophila* genome organization. Next, we describe emerging properties of the nuclear architecture and its functional significance based on our analysis of the model structure population.

#### Nucleolus and heterochromatin positioning

**Fig. 4:**
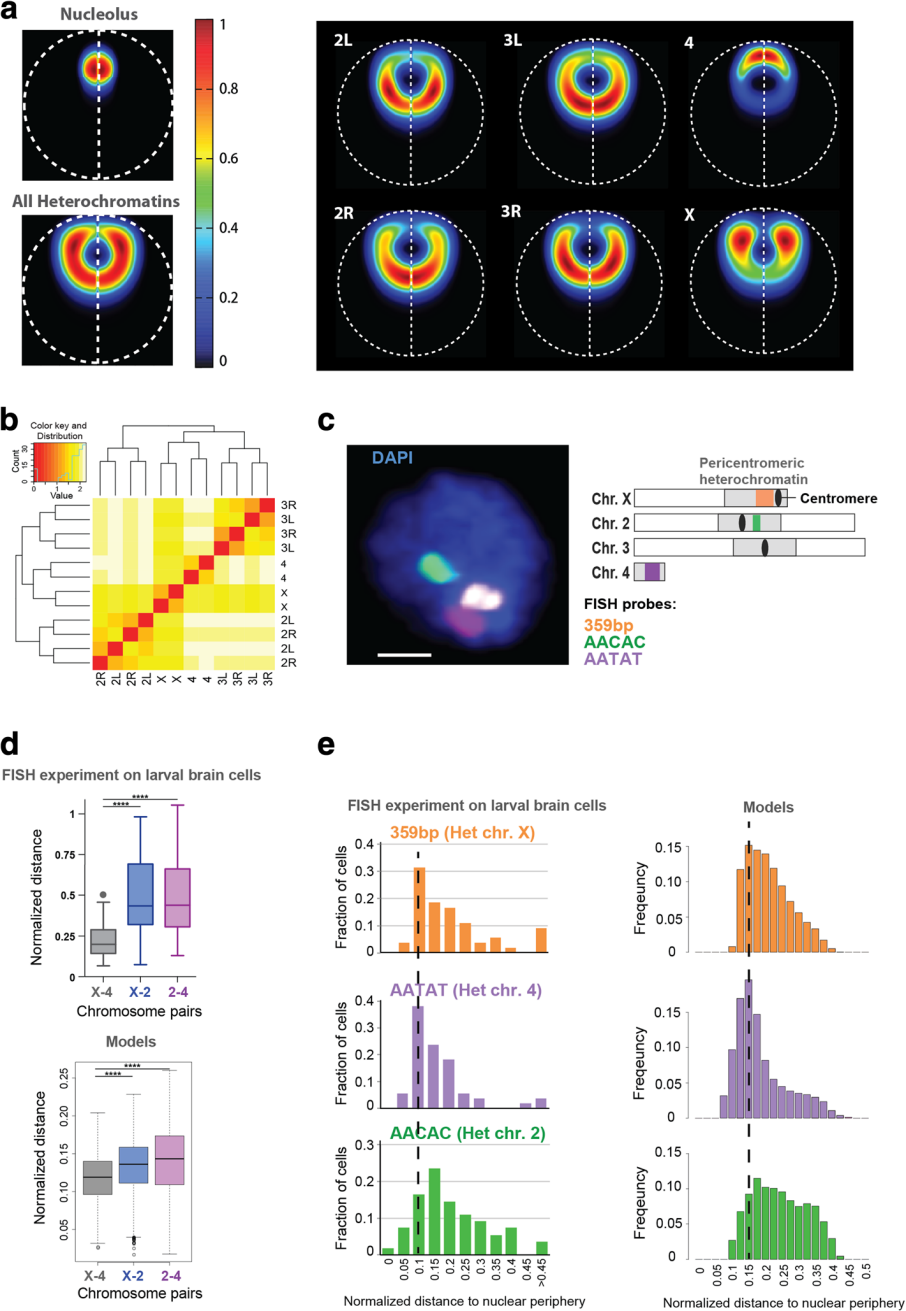
Heterochromatin and nucleolus positions. **a** *Left panel*: Localization probability density (LPD) plots of the nucleolus and all pericentromeric heterochromatin regions in the model. On average, the nucleolus occupies an intermediate position between the center and the periphery and is surrounded by pericentromeric heterochromatin. *Right panel*: LPD plots for pericentromeric heterochromatin of different chromosome arms. They all exhibit different preferred locations. Those of chr4 and chrX are significantly more peripheral than those of the other chromosomes. **b** Clustering of pericentromeric heterochromatin regions based on their averaged surface-to-surface distances. Heterochromatin domains of arms from the same chromosome naturally show preferred clustering. Heterochromatin domains from chr4 and chrX are usually closer to each other than to those from other chromosomes. **c** *Left panel*: FISH signals in larval brain cells. The image shows the middle Z-stack of a representative nucleus. Scale bar = 1 μm. *Right panel*: The position of FISH probes used for this study, relative to the pericentromeric regions of each chromosome (chrX, chr2, chr4). Note that the 359bp probe signal (*orange* in the scheme) is rendered in white in the FISH image. **d** *Top panel*: The positions (center-to-center distance normalized by the diameter of the nucleus) of heterochromatic satellites from different chromosomes relative to each other, measured in FISH experiments on larval brains; ****p value <0.0001 by paired *t*-test, N = 55 cells. *Bottom panel*: Pairwise distances (surface-to-surface distance normalized by the diameter of the nucleus) between the heterochromatin domains as measured in the model. Similar to the data in vivo, the distance between the heterochromatin domains of chrX and chr4 is significantly smaller than the distance between the other two pairs according to paired *t*-tests (*p* value <2.2e − 16). **e** *Left panel*: Positions of heterochromatic satellites from different chromosomes relative to the nuclear periphery, obtained from FISH experiments on larval brain cells. The heterochromatic satellites on chrX and chr4 are closer to the NE than those of chr2. *Right panel*: The distance from the center of heterochromatin to the NE normalized by the nuclear diameter as measured in the model. The models show a very good agreement with the experiment when considering the main trends, mainly: chrX and chr4 have higher histogram peaks located closer to the NE in comparison to chr2, and show a more focused localization probability towards the nuclear envelope. Note that the physical volume of the satellite repeats (imaged by FISH) is much smaller than the physical volume of the entire heterochromatin domain represented by a relatively large sphere in the model. This difference explains the offset observed at small distance values (i.e., starting at larger values) for the histograms, which corresponds to the radii of the corresponding spheres (i.e., 0.09, 0.05, and 0.08 normalized by nuclear diameter for chrX, chr4, and chr2R, respectively). For example, if a heterochromatin sphere is touching the NE, by definition the center distance to the NE is its radius. However, the satellite repeats that would be located inside the sphere could still be close to the NE

The nucleolus is a subnuclear structure linked to the assembly of ribosomal subunits. It is formed by nucleolar organizer chromatin regions (NOR), which contain the ribosomal DNA (rDNA) and are located close to the pericentromeric heterochromatin of chromosome X [65]. Our analysis allows the nucleolus to freely explore the nuclear space. However, the model predicts that the most likely radial position (on average) is in between the center and periphery of the nucleus (Fig. 4a, left panel; Additional file 1: Figure S4a), and that the large bodies of heterochromatin of each chromosome often enclose the nucleolus (Fig. 4a).

Importantly, we validated this model prediction in vivo using *Drosophila* Kc cells (Additional file 1: Figure S7). Immunofluorescence analysis of nucleoli and pericentromeric heterochromatin confirms that the average distance between the center of the nucleolus and the nuclear periphery is less than half of the nuclear radius (Additional file 1: Figure S7b). Interestingly, the nucleolus is positioned close to the nuclear periphery in 68% of cells, and close to the center of the nucleus in the remaining cells, revealing a bimodal distribution (Additional file 1: Figure S7a, c). In most cells, pericentromeric heterochromatin partially encloses the nucleolus (Additional file 1: Figure S7a).

Interestingly, our model predicts certain location preferences for pericentromeric heterochromatin of individual chromosomes. The heterochromatin regions of chromosomes 4 and X are more often close to each other (Fig. 4b; Additional file 1: Supplementary methods C.5), and both are more peripheral in the nucleus, than heterochromatin regions of chromosomes 2 and 3 (Fig. 4a, right panel). The model also predicts that chromosome 4 heterochromatin often tends to be positioned between the nucleolus and the NE (Fig. 4a, right panel; Additional file 1: Figure S4a). We reason that the metacentric chromosomes 2 and 3 are roughly double the size of the acrocentric chromosome X, and therefore they spread out more towards the interior of the nucleus. Notably, we confirmed these predictions using FISH staining of heterochromatic repeated sequences (satellites) in *Drosophila* cells of larval brains. As shown in Fig. 4c, the satellite repeats of chromosomes X and 4 are more often closer to each other than those of chromosomes X and 2, or 2 and 4 (Fig. 4d, top panel), in agreement with our models (Fig. 4d, bottom panel). Moreover, the satellite repeats of chromosomes X and 4 are more often closer to the nuclear periphery than those of chromosome 2 (Fig. 4e, left panel), which is confirmed by our findings in the model population (Fig. 4e, right panel). For example, the distribution for heterochromatin-NE distances of chromosome 2 is more widespread, containing a larger fraction of cells in which heterochromatin locations are further away from the NE in comparison to chromosomes X and 4 (i.e., the histogram in Fig. 4e of chromosome 2 is more widespread with a smaller maximum peak shifted towards larger distances in comparison to histograms of chromosomes 4 and X). All these features are well reproduced in our models. Together, these in vivo data support our model and suggest that the predicted chromosome organization is not limited to embryonic cells.

#### Localization of all euchromatin domains

**Fig. 5:**
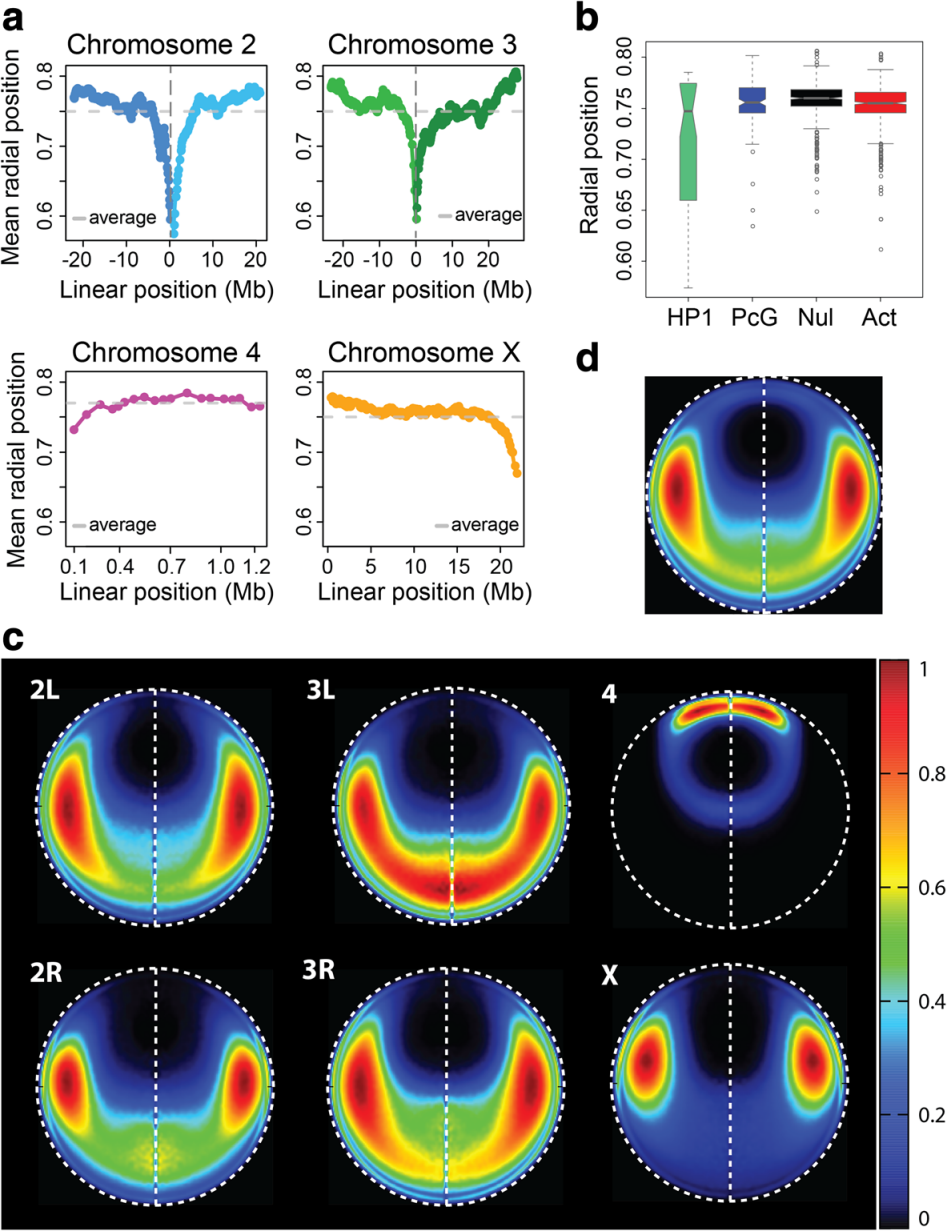
Localization of euchromatin domains in the structure population. **a** The average radial position for each euchromatin domain, plotted by position along its chromosome. The 0 location along the *x-axis* (*vertical dashed line*) of chr2 represents the euchromatin region closest to the centromere, with 2L domains on the left and 2R domains on the right. Chr3 domains are plotted with the same coordinate system as chr2. The domains of chr4 are plotted from *left* to *right*, while the domains of chrX are plotted from *right* to *left*; this convention follows the schematics in Fig. 1. Centromeric regions and pericentromeric heterochromatin regions are not shown in this figure. The domains near pericentromeric regions are closer to the nuclear center on average, while the domains near telomeric ends are preferentially close to the nuclear periphery. **b** The average radial positions of each domain, grouped by epigenetic class. **c** Localization probability density (LPD) plots of all euchromatin domains from each chromosome arm in nuclear space. **d** LPD plot of all euchromatin domains

When plotting the average radial position for every euchromatic TAD (Fig. 5a) we observe that the sequences near the pericentromeric heterochromatin are preferentially positioned in the nuclear interior, while euchromatic regions at the telomeric ends are more frequently proximal to the nuclear periphery. This preference is also seen for chromosome 4, despite its small size.

Euchromatic regions (excluding pericentromeric heterochromatin) are either active or repressed, and can be divided into four classes based on their epigenetic profiles: null, active, Polycomb-group (PcG), and HP1 [8] (Additional file 2: Table S3). The TADs of the null, active, and PcG classes have similar average radial positions (Fig. 5b). The average radial positions of the HP1 TADs have larger variance. The pericentromeric HP1 TADs (excluding all TADs on chr4) are found near the nuclear interior substantially more often than non-pericentromeric HP1 TADs.

Based on our model structures, we can create localization probability density plots (LPDs) for the euchromatic regions of different chromosomes (Fig. 5c). The chromosome with the most distinct location preference is number 4, whose euchromatic regions reside very close to the NE. In contrast, a large part of chromosome 3 L is located on the side of the NE opposite to chromosome 4 along the central axis, coinciding with the line drawn between the centers of the nucleus and nucleolus (vertical dashed line in Fig. 5c). Chromosome 2, on the other hand, prefers to avoid the central axis. The right and left arms have similar location preferences. The location distributions of chromosomes 2 and 3 are qualitatively similar, but chromosome 3 euchromatin is more likely to be found close to the central axis. Chromosome X euchromatin resides fairly close to the nucleolus, around the midpoint of the central axis, and is considerably less dispersed than the arms of chromosomes 2 and 3.

### Analysis of homologous pairing

#### Distances between homologous pairs vary along the chromosome

**Fig. 6:**
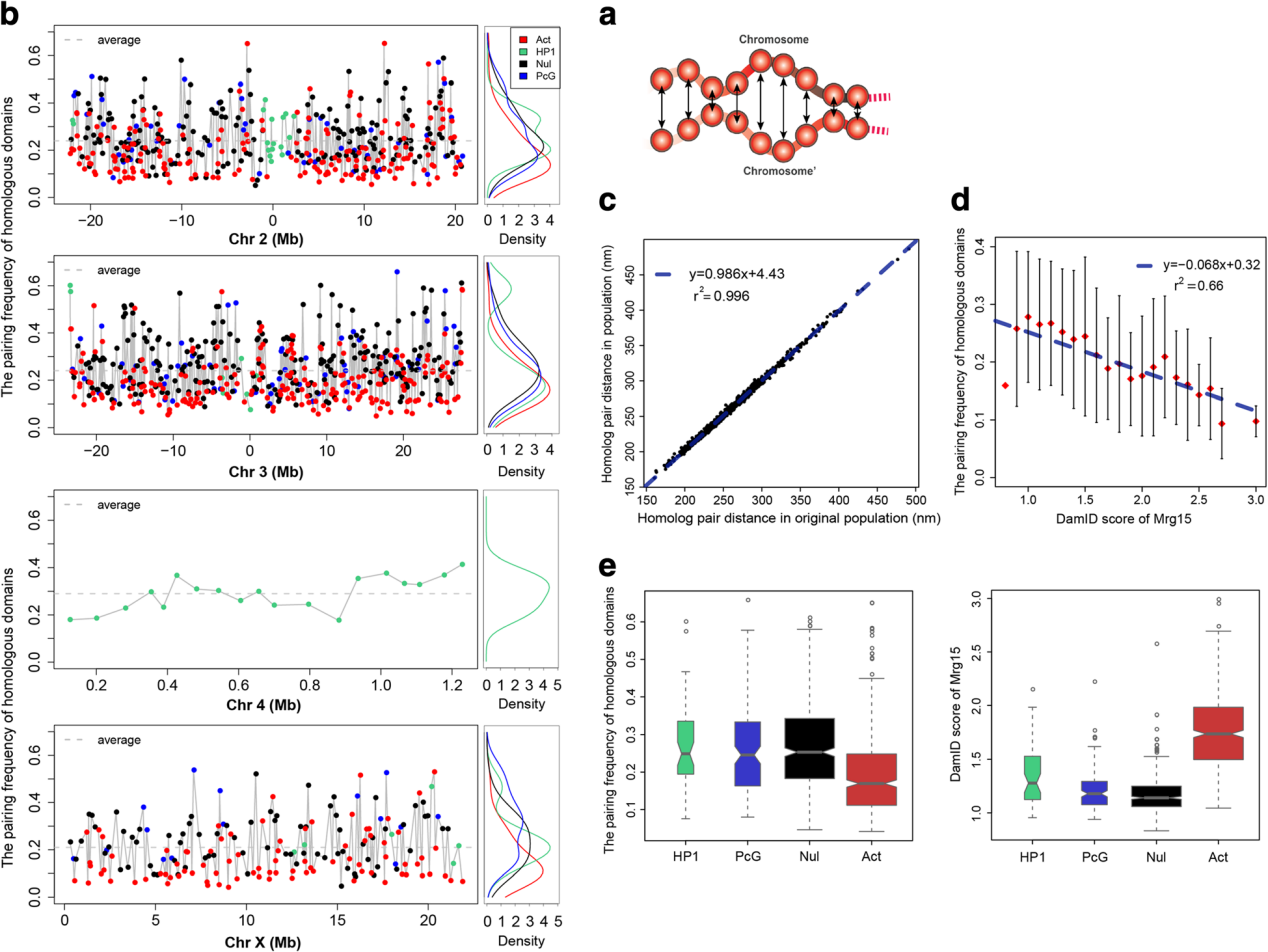
Analysis of homologous pairing. **a** Schematic view of surface-to-surface distances between homologous domains. Different domains exhibit different degrees of homolog pairing. **b** *Left panel*: Pairing frequency for each euchromatin domain, plotted by chromosome. We define a domain as being paired in a structure if the surface-to-surface distance between the two homologs is less than 200 nm. The *x-axes* are the same as the plots in Fig. 5a. The domains are colored by their epigenetic classes: *green*, HP1; *blue*, PcG; *black*, null; *red*, active. *Right panel*: Density plots of the domain pairing frequencies, grouped by epigenetic class. The active class has the smallest mean homologous pairing frequency for each chromosome. **c** Reproducibility of the average homolog distances between two independently generated structure populations. The Pearson’s correlation between them is 0.998, with *p* value <2.2e − 16. **d** The correlation between the pairing frequencies of homologous domains and their Mrg15 enrichment is negative. The Mrg15 scores range from 0.8 to 3.0 and are divided into 21 equal bins. The corresponding pairing frequencies from our models in a given Mrg15 bin are summarized as a mean and variance, and the latter is displayed as an *error bar*. The *blue dashed line* is the linear regression between the average pairing frequency in each bin and the midpoint Mrg15 enrichment value of the bin. The Pearson’s correlation between them is −0.81, with *p* value = 7.59-e − 06. **e** *Left panel*: Pairing frequencies of homologous domains grouped by epigenetic class. *Right panel*: Enrichments of Mrg15 binding grouped by epigenetic class. Active domains are generally more enriched with Mrg15, and have lower pairing frequencies, than the other three repressive classes

The genome of *D. melanogaster* is characterized by somatic homologous chromosome pairing in interphase nuclei [50, 52, 53, 64]. Moreover, the paired chromosomes touch only at a few specific interstitial sites [50]. In our structures, we define a domain as being paired if the surface-to-surface distance between the two homologs is less than 200 nm (Fig. 6a). Interestingly, the pairing frequencies of homologous domains show distinct and reproducible variation along the chromosomes (Fig. 6b, left panel), with the active class showing the lowest homologous pairing frequency for each chromosome (Fig. 6b, right panel). During the optimization, all pairs of homolog TAD copies are subject to a generic upper bound constraint, which limits their maximum separation to four times the TAD diameter. Even though this constraint is the same for all domains, in the optimized structures certain pairs of homolog TADs consistently have small average separations while others consistently have separations close to the upper bound. Hence, this distance variation is TAD-specific and highly reproducible in independently calculated structure populations (Fig. 6c). This effect is an indirect consequence of the genome-wide Hi-C and lamina-DamID constraints imposed on the structures.

The consistency of this pairing behavior raises the question of why certain regions attain higher levels of pairing. One clue is that we find a small but significant correlation between pairing frequency and the location of the TAD in the nucleus. Pearson’s correlation between the frequency of pairing and the frequency of being in proximity to the NE is 0.34 (*p* value <2.2e − 16; a TAD–NE contact is defined when its domain surface is less than 50 nm from the NE). We hypothesize that genomic regions that are often positioned near the NE may be more restricted in their movements, which may facilitate homolog pairing. We also investigated whether the local crowdedness around the domains could influence the spatial distances between homologs. We found that in most of the structures the local crowdedness is not different between paired domains and unpaired domains (Additional file 1: Supplementary methods C.6), suggesting that crowdedness does not affect pairing.

#### Mrg15 is enriched in active domains and depleted in repressive domains

Several proteins have been reported to affect somatic homolog pairing in *Drosophila* [52, 53, 64]. Among them is Mrg15, which binds to chromatin and recruits the CAP-H2 protein to mediate homolog unpairing [64]. Interestingly, we find an anticorrelation between Mrg15 binding enrichment and the frequency of homologous pairing in a domain, even though this information is not imposed as an input constraint in our models (Fig. 6d). The higher the Mrg15 enrichment in a domain, the lower the fraction of paired homologs in the structure population (Fig. 6d). Pearson’s correlation coefficient between the binned Mrg15 binding signal and the averaged frequency of homologous pairing for each bin is −0.81, with *p* value = 7.59e − 06 (Fig. 6d). In the control model (using only Hi-C data), the Pearson’s correlation coefficient between Mrg15 enrichments and pairing is −0.70 with *p* value = 4.46e − 4. We also divided the domains into three subsets based on their Mrg15 enrichment scores. The average pairing frequency of domains more enriched with Mrg15 is significantly less than that of domains with lower Mrg15 scores (one-tailed Mann–Whitney U test, *p* value <2.2e − 16; Additional file 1: Figure S8a).

Among the four TAD classes, active domains are generally more enriched with Mrg15-binding sites (Fig. 6e, right panel). Appropriately, we observe that transcriptionally active domains have a lower pairing frequency than the three repressive classes (Fig. 6e, left panel). The most intuitive explanation is that a loose pairing makes an active domain more accessible to regulatory factors. PcG domains, which are enriched with Polycomb group proteins, show higher levels of homologous pairing in our models than the active domains (one-tailed Welch’s two sample *t*-test, *p* value = 2.09e − 9). Therefore, our structure population supports the notion that PcG domains form tight pairs to enhance gene silencing (reviewed in [48]).

While active domains generally have low frequencies of homologous pairing, our models also have some clear and reproducible counterexamples of active domains with extremely high frequencies of homologous pairing (the specific TADs with this behavior are reproducible in independently generated structure populations; Fig. 6c). Therefore, we divided the active domains into two subclasses, labeled “active-tight” and “active-loose”. Interestingly, domains in the active-loose subclass have significantly higher Mrg15 enrichment than domains in the active-tight subclass (one-tailed Mann–Whitney U test, *p* value = 3.44e − 2; Additional file 1: Figure S8b). It is interesting that our model further supports a role for Mrg15 in disrupting homolog pairing, even though the structures were generated without any locus-specific constraint on the separation of homologous domains. Importantly, the anticorrelation between homolog pairing frequency and Mrg15 binding enrichment further increases when lamina-DamID data are integrated in the model, which indicates that data integration helps generate more accurate genome structures.

#### Active-tight domains show higher transcriptional efficiency

**Fig. 7:**
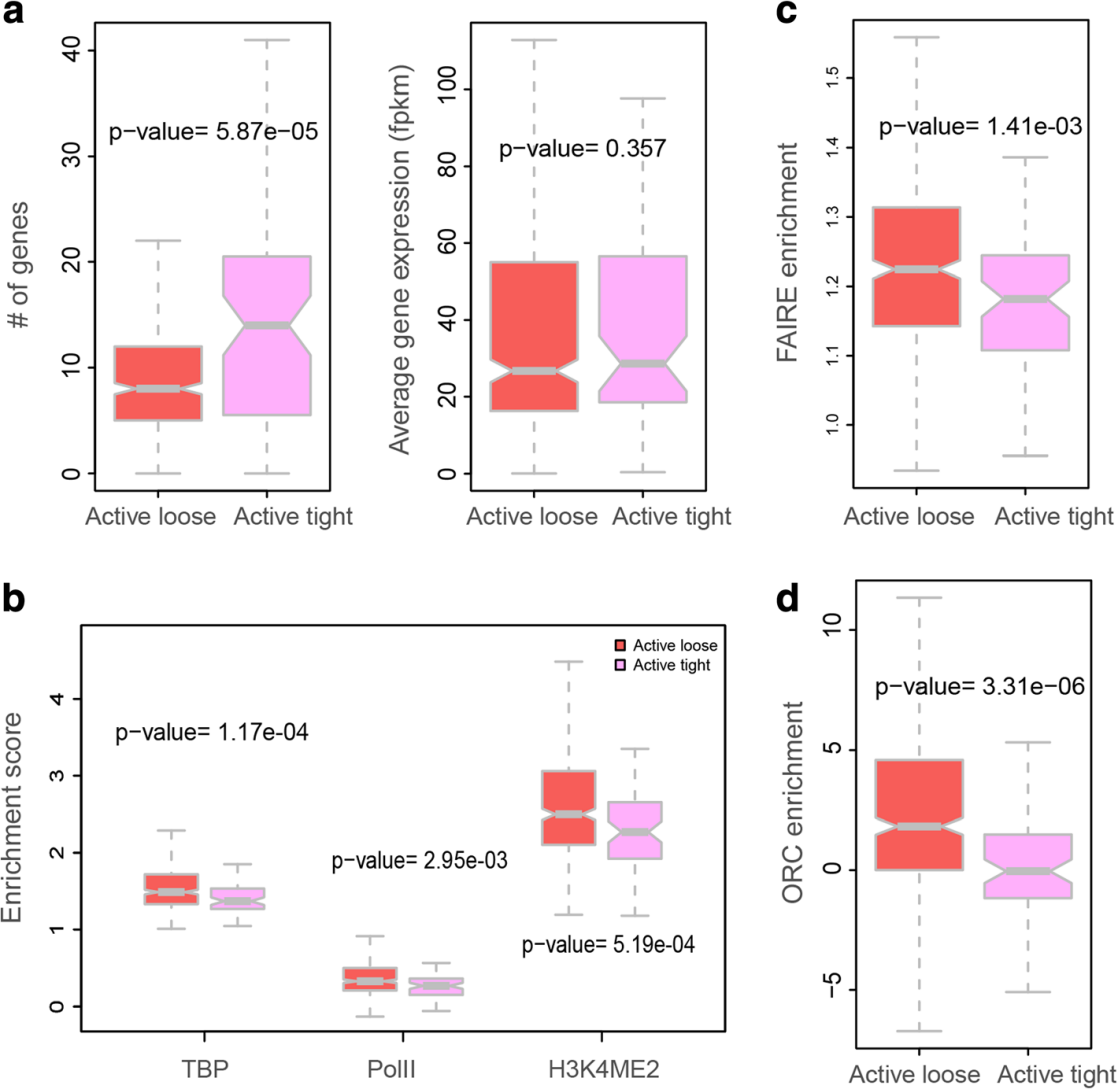
Transcriptional efficiency and DNA replication timing for genes in two subclasses of the active domains. **a** Domains in the “active-loose” subclass have lower frequencies of homolog pairing than those in the “active-tight” subclass (Additional file 1: Supplementary methods C.6). The active-tight subclass includes 71 domains and the active-loose subclass includes 423 domains. All the statistical tests were performed using one-tailed Mann–Whitney U test. *Left panel*: Domains in the active-tight subclass contain significantly more genes than domains in the active-loose subclass. *Right panel*: Genes in both subclasses have similar average expression values. **b** TBP (TATA binding protein), RNA polymerase II binding signal, and H3K4me2 signals are more enriched in domains of the active-loose subclass. **c** Formaldehyde-assisted isolation of regulatory elements (*FAIRE*) signal is significantly stronger in domains of the active-loose subclass. **d** Origin recognition complex (*ORC*) is significantly more enriched in domains of the active-loose subclass

Interestingly, we found significant functional differences between active-loose and active-tight domain subclasses. Active-tight domains contain more genes (Fig. 7a). Surprisingly, the active-tight subclass shows significantly lower binding levels of the TATA-binding protein (TBP) and RNA polymerase II, as well as lower H3K4me2 signals (one-tailed Mann–Whitney U test, *p* values 1.17e − 04, 2.95e − 03, and 5.19e − 04, respectively) (Fig. 7b). However, the gene expression levels in the two subclasses are comparable, despite the significantly smaller amount of bound RNA polymerase II transcription machinery in the active-tight subclass. This observation suggests that homolog pairing of active alleles might improve transcription efficiency even at lower concentration of transcription factors.

#### Active-tight domains tend to be later-replicating in comparison to active-loose domains

FAIRE (formaldehyde-assisted isolation of regulatory elements) is a biochemical method to identify nucleosome-depleted regions in the genome. It has been shown that these DNA sequences overlap with active regulatory sites and DNaseI hypersensitive sites [76]. Active-loose domains are significantly enriched with the FAIRE signal compared to domains in the active-tight subclass (Fig. 7c). This indicates that chromatin in the active-loose domains is more depleted of nucleosomes, and hence these domains contain a higher density of regulatory chromatin complexes. In *Drosophila*, the organization of nucleosomes plays an important role also in determining origin recognition complex (ORC) binding sites [77]. The difference in FAIRE enrichment led us to investigate DNA replication timing during interphase for the different classes. The active domains are generally more enriched with ORC than the other three types of domains, with significant *p* values (one-tailed Mann–Whitney U test, *p* values 3.26e − 15, 1.72e − 3, and 1.84e − 2 for null, HP1, and PcG, respectively), indicating that DNA replication is often initiated in the chromatin of the active class (Additional file 1: Figure S9b). In agreement, the active domains are generally more enriched with early origins of replication (defined in [77]) compared to null domains and PcG domains (one-tailed Mann–Whitney U test, *p* values 3.25e − 12 and 4.38e − 14, respectively), while overall HP1 domains (categorized as being part of the euchromatin areas) are more enriched in early origins (one-tailed Mann–Whitney U test, *p* value = 2.88e − 3) (Additional file 1: Figure S9c). This observation agrees with the strong positive correlation between early DNA replication and transcriptional activity reported for the *Drosophila* and human genomes [78, 79]. Strikingly, we discovered that both ORC-binding regions and early origins of replication are significantly more frequent in active-loose domains than in active-tight domains (one-tailed Mann–Whitney U test, *p* values 1.54e − 4 and 2.92e − 3, respectively; Fig. 7d; Additional file 1: Figure S9b, c), supporting the model that chromatin in the active-tight subclass replicates significantly later than chromatin in the active-loose subclass. Compared to other domain classes, active-tight domains show no significant difference in replication timing to null domains (*p* value = 0.13), but are replicated earlier than PcG domains (*p* value = 2.23e − 4) and replicated later than HP1 domains (*p* value = 1.78e − 4) (Additional file 1: Figure S9c).

## Discussion

It has become increasingly clear that a chromosome’s folding pattern and nuclear location have far-reaching impacts on the regulation of gene expression and other genome functions. Therefore, a thorough understanding of a genome’s function entails detailed knowledge about its spatial organization. A wide range of complementary technologies exist to provide such information. For instance, genome-wide ligation assays provide critical information about chromatin–chromatin interactions, lamina-DamID experiments reveal the propensity of a given locus to be located close to the NE, and 3D imaging technologies can reveal the spatial locations of individual loci in single cells. However, many computational models of genome structures rely on a single data type, such as Hi-C, which limits their accuracy. Integrating complementary data types increases the accuracy and coverage of genome structure models, and also provides a way to cross-validate the consistency of data obtained from complementary technologies. Thus, a major and vital challenge of computational biology is to develop hybrid methods that can systematically integrate data obtained from different technologies to generate structural maps of the nucleome (e.g., as this study integrates Hi-C and lamina-DamID data).

In this paper, we present a computational platform that can systematically integrate experimental data obtained from different technologies to map the 3D structures of entire genomes. Our probabilistic approach explicitly models the variability of genome structures between cells by simultaneously deconvolving data from Hi-C and lamina-DamID experiments into a model population of distinct diploid 3D genome structures. Our models therefore incorporate the stochastic nature of chromosome conformations, and allow a detailed analysis of alternative chromatin structure states.

Our method can be applied to genomes of any organism, including mammalian genomes. As a proof of principle, we mapped the structure of the *D. melanogaster* genome in interphase nuclei. We demonstrated that our method produces an ensemble of genome structures whose chromatin contacts are statistically consistent with Hi-C data while also reproducing the likelihoods of chromatin loci being close to the NE derived from lamina-DamID experiments.

The ensemble of model structures has strong predictive power for structural features not directly visible in the initial data sets. We observed that, in embryonic cells, chromosomes 2 and 3 are often organized with their centromeres and telomeres located in opposite hemispheres of the nucleus. In addition, each chromosome pair occupies a distinct territory in our models. Our structures also predicted correctly a relatively high colocalization probability between the two PcG-regulated Hox gene clusters.

Due to technical limitations, no Hi-C measurements are available to confirm interactions of repeated sequences, including most pericentromeric heterochromatin. However, using our 3D model structures, we can analyze the positions of chromatin loci with respect to pericentromeric heterochromatin. For instance, our model shows a high preference for the *white* gene on chromosome X to be positioned close to pericentromeric heterochromatin in comparison to similar gene locations on other chromosomes, thus facilitating the *white* gene’s translocation next to heterochromatin. Our analysis also reveals distinct differences between chromosomes in terms of heterochromatin localization probabilities. For example, pericentromeric heterochromatin of chromosomes X and 4 are more proximal to each other than to pericentromeric heterochromatin of chromosomes 2 and 3. The preferred euchromatin locations of chromosome 4 are also distinctly different from those of the other chromosomes.

We also make intriguing observations about homologous pairing that cannot be directly observed in the original Hi-C or lamina-DamID data. In our models, the tendency for domains to pair varies a great deal along the chromosome, which confirms the idea that pairing initiates from several distinct loci and spreads to neighboring regions. The observed pairing tendency of the domains is highly reproducible over several independent simulations, and also correlates with distinct functional features of the domains. We investigated why certain domains are more frequently paired than others. Interestingly, there is an anti-correlation between pairing frequency and the enrichment in Mrg15 protein binding, which is known to affect somatic chromosome pairing in *Drosophila*. This information was not explicitly included in the modeling process. The pairing frequencies of homologous domains also differ between those containing active or repressed chromatin. Active domains generally have a lower frequency of chromosome pairing than repressed domains such as those enriched in the Polycomb group (PcG) of proteins. However, we also identified some active domains that break this pattern, with extremely high rates of chromosome pairing across many independent simulations. Interestingly, when we compare these outlier active domains with the more common type of active domain having low pairing frequencies, the former have substantially lower levels of Mrg15 binding signals, later DNA replication timing, and lower FAIRE signals. These attributes are similar in other regions with high pairing frequencies.

Homologous pairing has been studied for years, and it has been found to play a large role in gene regulation. Transvection is a phenomenon whereby gene expression is modulated by the physical pairing of homologous loci. A case study showed that more transcripts are produced when both alleles of the gene Ubx are paired than when they are spatially separated [46]. A possible explanation is that each gene copy can be activated by both its own and the other copy’s enhancer [48]. Interestingly, when we compare actively transcribed genes in chromatin regions with very high or very low levels of homologous pairing, the former show significantly lower signals in RNA polymerase II and TATA protein binding, but at the same time similar levels of transcripts. This observation suggests that higher frequencies of pairing facilitate more efficient transcription of genes. Our model also shows that regions with looser homolog pairing initiate replication earlier than regions with tighter homolog pairing.

## Conclusions

In this study, we address one of the principal challenges of genome structure analysis: the development of a method that systematically integrates complementary data from different technologies to map the 3D organizations of genomes. Data from a single source, such as a Hi-C or lamina-DamID experiment alone, cannot capture all aspects of a genome’s organization. Integrating multiple data types is therefore not just beneficial but necessary to enhance the accuracy and coverage of structural models. Furthermore, the detailed analysis of such structural models is a valuable complement to experimental studies, because it can provide new structural insights. For example, the 3D models can reveal the relative locations of specific chromatin regions in the nucleus which are not immediately visible in the initial data. In the future, genome structure modeling should rely on all available data, including live fluorescence and 3D FISH imaging, as well as Hi-C and lamina-DamID experiments from both large-scale single cell and ensemble technologies. This approach will permit detailed analysis of the genome’s structural features, at high resolution and fully consistent with all experimental findings. Our work is a first step towards this goal, in that it allows the integration of genome-wide Hi-C as well as lamina-DamID data for 3D genome structure analysis, and provides a robust computational framework for integrating structural constraints from other types of experiments.

## Methods

### General description

The population-based approach is a probabilistic framework to generate a large number of 3D genome structures (i.e., the structure population) whose chromatin domain contacts are statistically consistent with experimental Hi-C data and other spatial constraints derived from a priori knowledge and/or independent data types. Our model is a deconvolution of the ensemble-averaged Hi-C data, and the resulting structures can be considered the most likely representation of the true structure population over a population of cells, given all the available data. Our method also distinguishes between interactions involving homologous chromosomes, so it can generate structure populations representing entire diploid genomes. Further, because the generated population contains many different structural states, this approach can accommodate all experimentally observed chromatin interactions, including those that would be mutually exclusive for a single structure. Compared to our previous research, which introduced the population-based approach using Hi-C data alone, in this study we also integrate lamina-DamID data to generate an improved structure population.

### Chromosome representation

The nuclear architecture of *Drosophila* cells consists of the nuclear envelope (NE), the nucleolus, and eight individual chromosomes (the diploid pairs chr2, chr3, chr4, and chrX). Chr2 and chr3 each have two arms, labeled 2L and 2R and 3L and 3R, connected by centromeres (Fig. 1b).

Each chromosome contains three main regions: euchromatin, pericentromeric heterochromatin, and a centromere (Fig. 1b). Euchromatin regions in chromosome arms 2L, 2R, 3L, 3R, 4, and X are linearly partitioned into a total of 1169 well demarcated physical domains [8], which are represented as spheres in the model [42]. A domain sphere is characterized by two radii: (1) its hard (excluded volume) radius, which is estimated from the DNA sequence length and the nuclear occupancy of the genome; and (2) its soft (contact) radius, which is twice the hard radius. A contact between two spheres is defined as an overlap between the spheres’ soft radii. This two-radius model allows for the possibility that chromatin can partially loop out of its bulk domain region to form contacts, while establishing a minimum genome occupancy in the nucleus. According to experimental data, the combined hard-core spheres of all euchromatin domains occupy around 12% of the nuclear volume. The total volume of heterochromatin is set to 1/27 of the nuclear volume. This figure is in agreement with estimates from microscopy images [66] (Additional file 1: Figure S7a), which show the heterochromatin cluster occupying roughly one-third of the nuclear diameter. The heterochromatin regions of each chromosome are modeled as spheres occupying volumes proportional to 5.4:11.0:8.2:8.2:3.1:20.0, according to the chromosome outlines depicted in Fig. 1b (these volumes are taken from the data shown in [80]). For every chromosome, the centromere is modeled as a sphere with 5% the volume of its corresponding heterochromatin domain (or sum of two heterochromatin domains for chr2 and chr3).

The nuclear radius is set to 2 microns (μm) as suggested by fluorescence imaging experiments [55, 66] (Fig. 4c; Additional file 1: Figure S7a). The nucleolus radius is set to one-sixth of the nuclear radius (Additional file 1: Figure S7a). Centromeres are clustered together and attached to the nucleolus [55]. Pericentromeric heterochromatin of chrX surrounds the rDNA cluster regions, so it lies in close proximity to the nucleolus. (Additional file 2: Table S3 lists all domain radii in the model).

All these units are represented by a total of 2359 spheres (Table 1).

**Table 1:** Structural units of our *D. melanogaster* genome model

Genome component	Unit quantity	Number of spheres	Description
TAD	1169	2338	Euchromatin TADs
HET	6	12	Heterochromatin clusters on 2L, 2R, 3L, 3R, 4, X
CEN	4	8	Centromeres of chromosomes 2, 3, 4, and X
Nucleolus	1	1	Localization of nucleoli

The outlines of the chromosomes are depicted in Fig. 1b. In the next section, we briefly describe the chromosome model and list all of the structural constraints that we imposed while optimizing the population.

### Probabilistic platform for data integration

Our method closely follows our recent population-based modeling framework [42]. However, we now generalize this framework to support the integration of lamina-DamID data with Hi-C data. The Hi-C data are contained in the ensemble contact probability matrix **A**, and the lamina-DamID data are contained in the ensemble chromatin–NE contact probability vector *E*.

We aim to generate a structure population *X* that maximizes the likelihood *P*(**A**, *E*|**X**). We introduce two latent variables *W* and **V**, which represent features of individual cells that aggregate into the ensemble information **A** and *E*, respectively. **W** = (*w*_*ijm*_)_2*N* × 2*N* × *M*_ is the contact indicator tensor, which contains the missing information in the Hi-C data **A**: the presence or absence of contacts between all domain homologs, in each structure of the population (*w*_*ijm*_ = 1 indicates a contact between domain spheres *i* and *j* in structure *m*; *w*_*ijm*_ = 0 otherwise). The second latent variable, **V** = (*v*_*im*_)_2*N* × *M*_, contains information on whether each domain homolog is located near the NE, in each structure of the population (*v*_*im*_ = 1 indicates that domain sphere *i* is near the NE in structure *m*; *v*_*im*_ = 0 otherwise). Note that while these latent variables are indexed over domain homologs (lowercase indices *i*, *j*), which are independent spheres in the model, the ensemble datasets **A** and *E* in the equations below are indexed over haploid domain identities observed in the experimental data (uppercase indices *I*, *J*). The maximum likelihood problem is then formally expressed as Eq. 1 and the expansion form is described as in Eq. 2.

Furthermore, *P*(**W**, **V**|**X**) can be expanded into a product of every contact indicator probability, i.e., $P\left(\mathbf{W},\mathbf{V}\left|\mathbf{X}\right.\right)={\prod}_{m=1}^M{\prod}_{\underset{i\ne j}{i,j=1}}^{2N}P\left({w}_{ijm}\left|{\overrightarrow{x}}_{im}\right.,{\overrightarrow{x}}_{jm}\right){\prod}_i^{2N}P\left({v}_{im}\left|{\overrightarrow{x}}_{im}\right.\right).$ Then the term *P*(**A**|**W**) can be expanded as *P*(**A**|**W**) = ∏_*I*,*J*_ *P*(*a*_*IJ*_|*a*'_*IJ*_) where *a*'_*IJ*_ is the contact probability of the domain pair *I* and *J*, $a{\prime}_{IJ}=\frac{1}{2M}{\sum}_{m=1}^M{\overline{w}}_{IJ m}$. The projected contact tensor $\overline{\mathbf{W}}={\left({\overline{w}}_{IJm}\right)}_{N\times N\times M}$ is derived from **W** by aggregating its diploid representation to the haploid counterpart.

Likewise, *P*(*E*|**V**) = ∏_*I*_ *P*(*e*_*I*_|*e*'_*I*_) , where *e*'_*I*_ is the probability for domain *I* to be near the NE. This is calculated as $e{\prime}_I=\frac{1}{2M}{\sum}_{m=1}^M{\overline{v}}_{Im}$. The term ${\overline{v}}_{Im}$ is a matrix element of the projected matrix $\overline{\mathbf{V}}={\left({\overline{v}}_{Im}\right)}_{N\times M}$ and indicates how many domain *I* representations in structure *m* are near the NE; thus, its possible values are {0, 1, 2} when the diploid representation is projected to the haploid counterpart.

With these probabilistic models, we can maximize the log-likelihood log *P*(**A**, *E*, **W**, **V**|**X**), expressed as follows:

5 $$ {\displaystyle \begin{array}{ll}\log \kern0.5em P\left(\mathbf{A},E,\mathbf{W},\mathbf{V}\left|\mathbf{X}\right.\right)\hfill & =\log \kern0.5em P\left(\mathbf{A}\left|\mathbf{W}\right.\right)+\log P\left(E\left|\mathbf{V}\right.\right)+\log P\left(\mathbf{W},\mathbf{V}\left|\mathbf{X}\right.\right)\hfill \\ {}\hfill & =\underset{N}{\overset{}{\sum_{I\ne J}^{I,J=1}}}\log \kern0.5em P\left({a}_{IJ}\left|a{\hbox{'}}_{IJ}\right.\right)+\sum \limits_{I=1}^N\log \kern0.5em P\left({e}_I\left|e{\hbox{'}}_I\right.\right)\hfill \\ {}\hfill & +\sum \limits_{m=1}^M\sum \limits_{\underset{i\ne j}{i,j=1}}^{2N}\log \kern0.5em P\left({w}_{ijm}\left|{\overrightarrow{x}}_{im},{\overrightarrow{x}}_{jm}\right.\right)+\sum \limits_{m=1}^M\sum \limits_{i=1}^{2N}\log \kern0.5em P\left({v}_{im}\left|{\overrightarrow{x}}_{im}\right.\right)\hfill \end{array}} $$

We assume that a pair of spheres (*i*, *j*) are in contact in structure *m* if and only if their center distance ${d}_{ijm}={\left\Vert {\overrightarrow{x}}_{im}-{\overrightarrow{x}}_{jm}\right\Vert}_2$ is between certain lower and upper bounds, *L* ≤ *d*_*ijm*_ ≤ *U*. The lower bound is the sum of their hard radii, *L* = *R*_*i*_ + *R*_*j*_, and the upper bound is the sum of their soft radii, *U* = 2(*R*_*i*_ + *R*_*j*_). We modeled the probability of a contact between two domain spheres *i* and *j* as a variant of the rectified or truncated normal distribution, expressed as:

6 $$ P\left({w}_{ijm}=1\left|{\overrightarrow{x}}_{im},{\overrightarrow{x}}_{jm}\right.\right)=\left\{\begin{array}{cc}\hfill 1,\hfill & \hfill L\le {\left\Vert {\overrightarrow{x}}_{im}-{\overrightarrow{x}}_{jm}\right\Vert}_2\le U\hfill \\ {}\hfill \exp \left(-,\frac{{\left({\left\Vert {\overrightarrow{x}}_{im}-{\overrightarrow{x}}_{jm}\right\Vert}_2-U\right)}^2}{2{\sigma}_w^2}\right),\hfill & \hfill {\left\Vert {\overrightarrow{x}}_{im}-{\overrightarrow{x}}_{jm}\right\Vert}_2>U\hfill \end{array}\right. $$

with very small variance, e.g., *σ*_*w*_ → 0.

The probability for a domain to reside near the NE is described as:

7 $$ P\left({v}_{im}=1\left|{\overrightarrow{x}}_{im}\right.\right)=\left\{\begin{array}{cc}\hfill 1,\hfill & \hfill {\left\Vert {\overrightarrow{x}}_{im}\right\Vert}_2\ge \lambda {R}_{\mathrm{nuc}}\hfill \\ {}\hfill \exp \left(-,\frac{{\left({\left\Vert {\overrightarrow{x}}_{im}\right\Vert}_2-\lambda {R}_{\mathrm{nuc}}\right)}^2}{2{\sigma}_v^2}\right),\hfill & \hfill 0\le {\left\Vert {\overrightarrow{x}}_{im}\right\Vert}_2\le \lambda {R}_{\mathrm{nuc}}\hfill \end{array}\right. $$

where *λ* = 0.975 to ensure that the enforced TAD is at the inside surface of the NE, and likewise *σ*_*v*_ → 0.

### Additional spatial constraints for the *Drosophila* genome

In addition to the data from Hi-C and lamina-DamID experiments, we include the following additional information as spatial constraints:

1. *Nuclear volume constraint*: All 2359 spheres are constrained to lie completely inside a sphere with radius *R*_nuc_, i.e., ${\left\Vert {\overrightarrow{x}}_{im}\right\Vert}_2\le {R}_{\mathrm{nuc}}$. Without loss of generality, we use the origin (0,0,0) as the nuclear center, so ${\left\Vert \overrightarrow{x}\right\Vert}_2$ is the distance from the nuclear center.
2. *Excluded volume constraint*: The model prevents any overlapping between the 2359 spheres, as defined by their hard radii. For every pair of spheres *i* and *j* in every structure *m*, we enforce ${\left\Vert {\overrightarrow{x}}_{im}-{\overrightarrow{x}}_{jm}\right\Vert}_2\ge \left({R}_{im}+{R}_{jm}\right)$.
3. Homolog *pairing constraint*: Based on experimental evidence, homologous chromosomes are somatically paired in *Drosophila* and so both copies of a gene are usually close to each other [50–53]. Therefore, we constrain the distance between two homologous domains to be less than an upper bound, which is four times the sum of their radii, i.e., ${\left\Vert {\overrightarrow{x}}_{im}-{\overrightarrow{x}}_{i\hbox{'}m}\right\Vert}_2\le 4\left({R}_i+{R}_{i\hbox{'}}\right)$.
4. *Consecutive TAD constraint:* To ensure chromosomal integrity, we apply an upper bound to the distance between two consecutive TAD domains, which is derived from the experimentally determined contact probability *a*_*ij*_. The upper bound distance is $$ {d}_{ij}\left({a}_{ij},{r}_i,{r}_j\right)={\left(\raisebox{1ex}{$7$}\!\left/ \!\raisebox{-1ex}{${a}_{ij}$}\right.+1\right)}^{\raisebox{1ex}{$1$}\!\left/ \!\raisebox{-1ex}{$3$}\right.}\left({r}_i+{r}_j\right) $$. Note that *d*_*ij*_ = 2(*r*_*i*_ + *r*_*j*_) when *a*_*ij*_ = 1.
5. *Additional knowledge-based chromosome integrity constraints*: The heterochromatic region of a given chromosome or chromosome arm forms a clustered subcompartment, so is represented by a single domain. No Hi-C data are available for the heterochromatic regions. To ensure chromosome integrity, the domains representing heterochromatic regions are always in contact with their adjacent TAD as well as with the centromeric domain. The constraint between the heterochromatin sphere and the adjacent TAD sphere *i* is ${\left\Vert {\overrightarrow{x}}_{Hm}-{\overrightarrow{x}}_{im}\right\Vert}_2\le 1.5\left({R}_H+{R}_i\right)$. The constraint between the heterochromatin domain and the adjacent centromere sphere is ${\left\Vert {\overrightarrow{x}}_{Hm}-{\overrightarrow{x}}_{Cm}\right\Vert}_2\le 1.1\left({R}_H+{R}_C\right)$, where ${\overrightarrow{x}}_{Hm}$ and ${\overrightarrow{x}}_{Cm}$ are the centers of the heterochromatin and centromere spheres, and *R*_*H*_ and *R*_*C*_ are the hard radii of the heterochromatin and centeromere spheres. Based on experimental evidence [55], all centromeres are in proximity to the nucleolus. Therefore, we constrain the centromere spheres to be close to the spherical volume representing the nucleolus, defined as ${\left\Vert {\overrightarrow{x}}_{Nu}-{\overrightarrow{x}}_C\right\Vert}_2\le 1.1\left({R}_{Nu}+{R}_C\right)$, where *R*_*Nu*_ is the radius of the nucleolus volume.

### Distance threshold method for estimating **W** and **V**

We adopt the distance threshold method introduced elsewhere [42] to estimate the distribution of contacts among the diploid genome across a population of structures. The distance threshold *d*_*IJ*_^*act*^ for each domain pair (*I*, *J*) is determined based on the empirical distribution of all distances between their homologous copies across all structures of the population. The procedure to determine a distance threshold for estimating an element of the projected contact indicator tensor, ${\overline{w}}_{IJm}$ , is as follows. Let (*I*, *J*) be a domain pair (with homologs *i*, *i’* and *j*, *j’*) and let their Hi-C contact probability *a*_*IJ*_ > 0. We construct an empirical distribution of the pairwise domain distances between homologous copies of the domain pair (*I*, *J*). When *I* and *J* are domains from the same chromosome, we collect the distances *d*_*ijm*_ and *d*_*i*'*j*'*m*_ in all model structures (*m* = 1, 2, …, *M*), forming a set of 2 *M* distances. When *I* and *J* are domains from different chromosomes, we collect the smallest two distances from the set of all possible distances {*d*_*ijm*_, *d*_*i*'*jm*_, *d*_*ij*'*m*_, *d*_*i*'*j*'*m*_}, again for a total set of 2 *M* distances. Next, the 2 *M* distances are ranked in increasing order. The distance threshold, *d*_*IJ*_^act^, is defined as the distance value with the (2 *M* ⋅ *a*_*IJ*_)th rank among the 2 *M* sorted distances. Once all the distance thresholds are obtained, we populate the tensor $\overline{\mathbf{W}}$ by counting how many of the pooled distances between (*I*, *J*) from structure *m* in the set of 2 *M* distances that fall below the corresponding distance threshold. The structure optimization then assigns contacts to the pairs with shorter distance out of four possible pairs between homolog domains, for every *w*_*ijm*_. This procedure maximizes log *P*(**A**, **W**|*X*), which is composed of two items: log *P*(**W**|**X**) and log *P*(**A**|**W**). This is true for two reasons: (i) it assigns contacts only to domain pairs with short distances, maximizing log *P*(**W**|**X**); and (ii) it uses the 2*a*_*IJ*_ *M* ^th^-quantile of all 2 *M* distances as the distance threshold to determine *w*_*ijm*_, which heuristically maximizes the first term $\log P\left(\mathbf{A}\left|\mathbf{W}\right.\right)=\sum_{\underset{I\ne J}{I,J=1}}^N\log P\left({a}_{IJ}\left|{a}_{IJ}^{\hbox{'}}\right.\right)$ by making *a*_*IJ*_ exactly equal to *a*_*IJ*_^'^.

We adapted this procedure to estimate the TAD–NE contact matrix **V** = (*v*_*im*_)_2*N* × *M*_. The distance threshold for every TAD is determined. Again we sort a set of 2 *M* distances to the NE related to domain *I* in increasing order, and select the (2 *M* ⋅ *e*_*I*_)th rank as the distance threshold. Once the distance thresholds are obtained, we populate the matrix $\overline{\mathbf{V}}={\left({\overline{v}}_{Im}\right)}_{N\times M}$ by counting how many of the pooled distances from each structure *m* in the 2 *M* distances are lower or the same as the corresponding distance threshold. Note that there are only three possible values of the matrix element: ${\overline{v}}_{Im}\equiv \left\{0,1,2\right\}$. A value of 2 means that both homologs of TAD have to be located near the NE; a value of 1 means only one of the homologs has to be located near the NE; and a value of 0 means that neither homolog is forced to be located near the NE. The optimization step will then assign *v*_*im*_ accordingly as either 0 or 1. When ${\overline{v}}_{Im}=1$, the ambiguity as to whether (*v*_*Im*_ = 1, *v*_*i* ' *m*_ = 0) or (*v*_*im*_ = 0, *v*_*i* ' *m*_ = 1) is solved on the fly, during the dynamic optimization of the genome structure, where 1 is favored for shorter distances to the NE.

### Optimization

As described elsewhere [42], we used step-wise optimization and the A/M iteration algorithm to generate the structure population. We first generated a population of structures satisfying all Hi-C constraints, then fine-tuned the model structures by gradually including the lamina-DamID constraints. For the Hi-C constraints, we included new contact probabilities in several stages during the optimization, at the lower thresholds **Θ** = {1, 0.7, 0.4. 0.2, 0.1, 0.07, 0.06}. One or more iterations were performed at every probability level. Contact probabilities less than 0.06 were not used at all. Twenty-six A/M iterations were required to generate a structure population consistent with the Hi-C data. The lamina-DamID data were also included in several stages, at the probability levels **Θ** = {0.2, 0.1, 0.06}. Ten additional A/M iterations were performed to optimize the structure population with respect to the lamina-DamID data. The optimization was performed using a combination of simulated annealing molecular dynamics and conjugate gradient methods. The algorithm was implemented using the Integrated Modeling Platform (IMP) [81].

### Data collection and processing

Our processing methods for Hi-C, lamina-DamID, and other epigenetics data are described in Additional file 1.

### Analysis of the structure population

Our statistical analysis of the structure population and details on all statistical tests are described in Additional file 1.

### Robustness analysis

We tested the robustness of our modeling approach in four tests: (i) replicate simulations, (ii) variation of population size, (iii) variation of homolog-pair upper-bounds, and (iv) variations of input domain contact probability. The details are described in Additional file 1. In conclusion, we can show that varying all relevant parameter settings does not significantly affect the conclusions of the paper. All results are highly reproducible under the variation of these parameter settings.

### Cell culture and immunofluorescence

Drosophila Kc cells were maintained at 27 °C as logarithmically growing cultures in Schneider's medium (Sigma) + FBS (Gemini), and fixed and stained as previously described [66]. Antibodies used were anti-Fibrillarin (Cytoskeleton, catalog number AFB01; 1:200) and anti-H3K9me2 (Upstate, catalog number 07-442; 1:500).

### FISH

Wild-type *w* ^*1118*^ flies were raised at 25 °C. Brains were dissected from third instar larvae and squashed before fixation, as described in [82]. Fixation and FISH staining were carried out as described in [83], using the following probes: 5′-6-FAM-(AACAC)_7_ for chromosome 2 satellites, 5′-Cy3-TTTTCCAAATTTCGGTCATCAAATAATCAT for chromosome X satellites (359 bp), and 5′-Cy5-(AATAT)_6_ for chromosome 4 satellites. FISH probes were purchased from Integrated DNA Technologies, and designed as described in [82].

### Imaging and image analysis

All images were captured using a Deltavision fluorescence microscopy system equipped with a CoolsnapHQ2 camera, using 60× and 100× objectives and 10–12 Z stacks with Z-intervals of 0.2–0.4. Images were deconvolved with softWorx software (Applied Precision/GE Healthcare) using the conservative algorithm with five iterations. The distances between signals in 3D volume reconstructions of Kc cells or in individual Z stacks of larval tissues were calculated with softWorx. All distances were normalized to the nuclear diameter of their respective cells. Quantification of FISH signals in larval brain squashes was limited to cells that displayed clear homologous pairing, defined as proximal or overlapping FISH signals for each probe.

## Additional files

Additional file 1: Supplementary methods A–D and Supplementary **Figures S1–S14**. (DOCX 1764 kb)

Additional file 2: Three supplementary tables, each included as a separate tab. **Table S1.** Summary of the Pearson’s correlation between contact probability from structure models and Hi-C experiment. **Table S2.** Summary of chromosomal territory index (TI) for individual arms and pairs of homologous arms. **Table S3.** The sphere size of structural units of model. (XLS 131 kb)
