# Supplementary material for: The three-dimensional genome organization of Drosophila melanogaster through data integration
Source: Genome Biol. 2017 Jul 31;18:145. doi: 10.1186/s13059-017-1264-5 (PMC5576134; doi:10.1186/s13059-017-1264-5)
Supplement: Supplementary file 1 — Supplementary methods A–D and Supplementary Figures S1–S14. (DOCX 1764 kb) [file 13059_2017_1264_MOESM1_ESM.docx]

#### Supplementary methods

# Hi-C data processing

## Bin-level contact frequency

The sequencing data were downloaded from Gene Expression Omnibus under accession number GSE34453 [[1](#_ENREF_1)]. We adopted the pipeline developed by Leonid Mirny lab [[2](#_ENREF_2)] to process the Hi-C data. First, the two sides of each read were mapped to the *Drosophila melanogaster* genome (assembly dm3) independently using bowtie2 [[3](#_ENREF_3)] with “very-sensitive” option. We truncated the reads to 20bp, and then remapped the non-mapped and multiple mapped reads by increasing truncation length with 5bp gradually. The truncating step significantly yields more double-sided mapped reads. 216,199,696 uniquely double-sided mapped reads are retained from the original 362,669,793 paired reads (with the mapping ratio at ~60%).

Then, the reads alignments for the artificial or non-informative contacts were filtered out, including self-ligation products, the products without ligation junction and PCR duplicates. After filtering process, 14,481,367 interactions are left for downstream analysis. Third, the valid double-sided alignments were used to construct the genome-wide contact matrix at 40k bin size, resulting in a 3012*3012 matrix.  Before correcting the matrix for biases, we performed four types of bin-level filtering which will affect the normalization procedure. We removed the contacts between loci located within the same bin; removed bins with more than 50% are N’s in the reference genome; removed 1% of bins with low coverage; and truncated top 0.05% of inter-chromosomal counts which truncated values of the top 0.05% to be the same as highest value among the rest 99.95%. Finally, the iterative correction was performed on the filtered genome-wide contact map to get a normalized map, denoted as $F_{K}={(f_{ij})}_{K*K}$.

## Bin-level contact probability

The contact probability is defined as the probability for observing a given contact in the structure population. We define a threshold value $f^{max}$, which defines the frequency at which a contact is formed in 100% of the structure population. We assume the contact frequencies that constitute any stable TAD can serve as reference where the interaction could exist in 100% of the cells. First, we register all contact frequencies inside TADs. Then we apply an R (CRAN statistical software) function *boxplot.stats* on this contact frequency set to get the extreme lower whisker of the boxplot and set it as the $f^{max}$. Namely, in R

$$f^{max}=boxplot.stats\left( f_{ij}(intra TAD) \right) \$stat[1]$$

The contact probability between bin i and bin j is derived as

$$a_{ij}=\min\{\frac{f_{ij}}{f^{max}}, 1\}$$

We applied the $f^{max}$ calculation method to the normalized frequency matrix to obtain the contact probability at 40 kb-binned matrix.

## Domain-level contact probability

Based on the Hi-C contact frequency map, 1169 physical domains or topological associated domains (TADs) are detected by a quantitative probabilistic approach [[1](#_ENREF_1)]. The chromatin in our population structure is represented at the level of TADs, therefore the contact probability at domain-level need to be obtained. The domain level contact probability is denoted as $A_{N}={(a_{IJ})}_{N*N}$, where $a_{IJ}$ is the contact probability between domain $I$ and domain $J$, and $N$ is the total number of domains in the genome. $a_{IJ}$ is derived from the corresponding contact probability at the bin level. If $b(I)$ is the set of all bins in domain $I$, and $b(J)$ is the set of all bins in domain $J$, then

$$a_{IJ} = mean \left\langle\left. top 10\%\left\{ a_{ij} \right.\left. | i \epsilon b\left( I \right), j \epsilon b\left( J \right) \right\} \right\rangle\right.$$

is the average value of the top 10% ranked contact probabilities in the set of all pairwise combinations between bins in $b(I)$ and $b(J)$.

# Lamina-DamID data processing

The genome-wide lamina-DamID binding signal is collected from [[4](#_ENREF_4)]. The binding signal for each TAD ($L=\{l_{I}|I=1,2,...,N\}$) is calculated using BigWigSummary tool (from USC Genome Browser). The DNA content of the nuclear periphery was measured to be ~12% per nucleus for Kc167 cell line [[5](#_ENREF_5)]. To reproduce the experimentally observed DNA content at the nuclear periphery, we relate the average lamina-DamID signal of all chromatin regions (measured in an ensemble of cells) to the average domain-NE localization probability () in the structure population so that $\text{mean(}E\text{)=0.12}$. The lamina-DamID signal is transferred into a probability for each TAD to be close to the NE as $\text{e}_{I}=\frac{0.12}{\text{mean}(L)}l_{I}$.

# Analysis of the structure population

## Reproducing lamina-DamID binding frequency

We define a chromatin domain-lamina contact, if the distance between the domain’s surface and the NE is less than 50nm. The domain-NE association probability is the fraction of structures in the population, in which the domain is in contact to the NE.

## Chromosome territory index

To quantify how effectively one chromosome excludes other chromosomes from the volume it occupies in the 3D space, we adopted the quantity called chromosome territory. There is no universal definition of chromosome territory, but we follow the definition in a recent publication about the structure modeling of Drosophila polytene chromosomes [[6](#_ENREF_6)]. Chromosome territory index (TI) is defined as the fraction of domains inside a convex hull that belongs to the chromosome used for its construction.

We first calculate the convex hull for a chromosome arm (with domain number $N_{chr}$) using the function *delaunayn* in MATLAB (http://www.mathworks.com/help/matlab/ref/delaunayn.html). *T=delaunayn(X)* computes a set of simplices such that no data points of X are contained in any circumspheres of the simplices. The set of simplices forms the Delaunay triangulation. Then, the function *tsearchn* is used to search all the domains inside of the convex hull, and the number of detected domains is denoted as $N_{hull}$. Finally, the TI is calculated as $\frac{N_{chr}}{N_{hull}}$.

The maximum $N_{hull}$ for each chromosome are the same, which is the total number of domains inside the nucleus (2238 euchromatin TADs in this study). The minimum $N_{hull}$ for each chromosome corresponds to the number of domains that belongs to themselves respectively. Under this definition, the maximum TI is 1, indicating that all domains inside the chromosome spanning volume are exclusively occupied by its own chromosome domains, and therefore shows a strong chromosome territory formation with only limited chromosome mixing. The theoretical minimum values for each chromosome arm are 0.096, 0.091, 0.095, 0.131, 0.008 and 0.0787, and for each pair of homologous arms are 0.192, 0.182, 0.189, 0.263, 0.016 and 0.157. The average TIs for chromosome arms in the structure population are 0.64 (2L), 0.65 (2R), 0.62 (3L), 0.62 (3R), 1.0 (4) and 0.67 (X). The average TI for individual arms is around 60%, suggesting the homolog pairs share territory almost equally. Indeed, the paired arms together possess high territorial index, i.e. 0.97, 0.98, 0.96, 0.98, 1.0 and 0.98 for arms 2L, 2R, 3L, 3R, 4, and X, respectively.

## Residual polarized organization

The polarized (Rabl-like) organization shows that each chromosome occupies an elongated territory, with the centromere in one nuclear hemisphere and telomere in the opposite hemisphere. We investigated the position of each centromere and its corresponding telomere and obtain the number of polarized configuration chromosomes or arms (chr4 are excluded, therefore the number ranges from 0 to 10). To identify the presence of this organization, we measure the angle between each centromere, the nuclear center, and its corresponding telomere. If the cosine of the angle is positive, then centromere and telomere are in the same hemisphere. Otherwise, they occupy opposite hemispheres, forming polarized organization. If more than half of the chromosome arms (>=6) in one nucleus are in polarized organization, we consider this as a polarized nuclear structure.

## Nuclear colocalizations of Hox gene clusters

The Antennapedia complex contains 5 genes located in 3 consecutive TAD-s, while the Bithorax complex contains 3 genes located in 2 TAD-s with one domain between them. Each control group contained two clusters, one cluster with 3 consecutive repressive domains and the other with 2 repressive domains separated by one domain. The 5 repressive domains were separated by the same linear distances as those in the Hox gene clusters. Because there are no available combinations of PcG domains with the same genomic distances as the Hox gene clusters, the control data set involved the three types of repressive classes (Null, PcG and HP1 class). In total, we identified 30 combinations that meet the requirements of control groups.

## Pericentromeric heterochromatin cluster detection

We calculated all pairwise surface-to-surface distances (normalized by the sum of the radii of the domain spheres) among the 12 heterochromatin spheres, for each structure in the population, then obtained the average pairwise distances in the matrix. Hierarchical cluster analysis is performed on the average distance matrix by using *hcluster* function in R.

## Homologous pairing

A domain is defined as paired if the surface-to-surface distance between two homologs is less than 200 nm. Then the pairing frequency is defined as how often a domain is paired among the structure population. The domains with pairing frequency higher than Top 3^rd^ quantile among all the domains are determined to be “tight”, otherwise, to be “loose”. We have 71 “active-tight” domains and 423 “active-loose” domains.

The variation of the distance along homologous chromosomes and among nuclei raises the question of why certain regions attain higher level of homologous pairing than others in certain nuclei. First, we notice that the linear distance to centromere or to heterochromatin does not influence the extent of pairing of a domain (**Fig. 6B**), which exclude a possible influence of heterochromatin clustering on the extent of pairing. Next, we tested whether the 3D position of a domain in the nucleus influences pairing. We hypothesize that genomic regions near NE may have less space for movement, thus promoting homologous pairing. Indeed, the Pearson’s correlation between the contact frequency with NE (if the surface distance to NE is less than 50 nm, this domain is defined in contact with NE) and the homologous pairing frequency is 0.34 with p-value < 2.2e-16. Similarly, Pearson’s correlation between the average radial position and the homologous pairing frequency is 0.10 with p-value = 4.9e-4. Finally, we tested the hypothesis that the crowdedness of the neighborhood around a domain influences this domain’s pairing. The Neighborhood Crowdedness (NC) of a domain is defined as the number of other domains whose surface-to-surface distance to the domain is less than 200 nm. We calculate NC for each pair of homologous domains in individual models and compare the difference between paired and unpaired groups. The domains of paired groups have higher NC than the unpaired in 16.62% of models and lower in 6.84%, the rest of models (76.54%) show no significant difference. This data support the idea that the NC around domains does not significantly influence the pairing of homologous regions.

## Epigenetic analyses

Chromatin domains were classified into four classes based on their epigenetic signatures: Active, Polycomb-Group (PcG), HP1/Centromere and Null [[1](#_ENREF_1)]. Active domains comprise 42% of the domains with smaller domain size, and they are actively transcribed and characterized by high gene density. PcG domains are bound by PcG proteins and associated with the histone mark H3K27me3. HP1/Centromere domains are bound by the heterochromatin proteins HP1 and Su(var)3-9 and associated with H3K9me2. Null domains are not enriched for any of those marks.

We followed this 4-class annotation in our structure analysis. We also collected the data of histone modifications and binding of chromatin proteins in the study [[4](#_ENREF_4)] from <http://research.nki.nl/vansteensellab/Drosophila_53_chromatin_proteins.htm>. Wig files were downloaded, and then transferred into bigwig format. BigwigSummary program (from USC Genome Browser) was used to extract the individual signals for requested regions (the defined 1169 TADs in this study). The signal was calculated as an average per domain, avoiding the bias of the genomic length.

## Transcription analyses

Gene expression data (embryonic samples collected at 16-18h) were obtained from the modENCODE project [[7](#_ENREF_7)]. 1169 physical domains covered 12947 genes with available expression data. The number of genes in each domain varies, ranging from 0 to 170, and the average number is 11. The average gene expression values are calculated for each domain. RNA polymerase II binding data for Kc167 cells were also from modENCODE project (accession no. GSE20806, link <http://www.ncbi.nlm.nih.gov/geo/query/acc.cgi?acc=GSE20806>). TBP (TATA-binding protein) is a component of the basal transcription machinery and the protein binding data are from [[4](#_ENREF_4)]. BigwigSummary program was used to calculate the average signal for each defined domain.

## DNA replication analyses

Data for ORC-binding regions and early activating replication origins for Kc167 cell line were downloaded from modENCODE (accession no. [GSE20889](http://www.ncbi.nlm.nih.gov/geo/query/acc.cgi?acc=GSE20889) and [GSE17285](http://www.ncbi.nlm.nih.gov/geo/query/acc.cgi?acc=GSE17285), respectively). BigwigSummary program was used to calculate the average signal for each defined domain.

## Statistical test

The association test between two paired signals is done by *cor.test* function in R using Pearson's product moment correlation coefficient. For example, the positive correlation between the frequency near NE derived from our population of structures and the lamina binding signal from lamina-DamID experiment provides a validation for our models; the negative correlation between the frequencies of homologous pairing derived from our population of structures and the Mrg15 protein binding signal from lamina-DamID experiment matches the unpairing function of Mrg15 protein and also validate our models.

The difference test between two sets is done by *wilcox.test* function in R, which performs a Wilcoxon rank sum test (equivalent to the Mann-Whitney test) when the population cannot be assumed to be [normally distributed](http://en.wikipedia.org/wiki/Normally_distributed). For example, we found the ORC binding signals are much stronger in the active-loose than in the active-tight domains.

# Robustness analysis

We tested the robustness of our modeling approach in four perspectives:

## Robustness with respect to independent replicate simulations

We generated independently calculated replicate simulations using the same input parameter setting but different random starting domain configurations. All our results are highly reproducible. For example, the average radial domain positions and homolog pair distances are very well reproduced between the replica simulations (**Fig. S10 A, B** and **Fig. 6C**).

## Robustness with respect to population size

We previously showed that a population size of 10,000 was sufficient to have highly convergent result [[8](#_ENREF_8)]. To demonstrate the robustness of our results with respect to the population size for the Drosophila genome, we now also generated a population with 3,000 structures by rerunning the pipeline, and compared our results with those generated with the initial population of 10,000 structures. All our results are reproducible with a population of 3,000 structures. The Pearson correlation (PCC) for the average radial position of euchromatin domains between the two populations is 0.977 (with p-value < 2.2e-16) and the linear regression is shown in **Fig. S11A**. The PCC for the average homolog domain distances between the two populations is 0.997 (p-value < 2.2e-16) and the linear regression is shown in **Fig. S11B**. All other reported results in the manuscript are also reproducible with the smaller population size of 3,000 structures, demonstrating that a population size of 3,000 structures has reached the required convergence for the features we analyze. To test further the robustness of other parameter settings we generated several other structure populations with size 3,000 (see D.3 and D.4 below).

## Robustness with respect to upper bound distance between homolog-pairs

We also analyzed a structure population when varying the upper distance bound between homolog domain pairs. The upper bound was choosen to be 4 times of the domain diameter (4X-model). A structure population was calculated by increasing this number by 25%, namely an upper bound of 5 times the domain diameter (5X-model). We found that all results and conclusions remain unchanged.

- 1. *Domain radial positions*. The average radial positions of all domains are essentially unchanged when varying this parameter (PCC for the radial position of euchromatin domains between two populations is 0.995 (p-value < 2.2e-16).
  2. *Homologue pairing frequency.* The PCC for the homolog distances between two populations is 0.956 with p-value < 2.2e-16. As expected, the average homolog distances for structures in the 5X-model are larger than the ones from the 4X-model (the average homologous distance is 333 nm versus 262 nm). Following our analysis protocol we define the distance cutoff for homologous pairing (using the surface-to-surface distance cutoff <= 252 nm, which is defined as < ~75% of the average homogous pair distance) to define pairing. The conclusions all hold and indeed some results are even more significant.

**First**, the negative correlation between the pairing frequency of homologous domains and Mrg15 enrichment is maintained with PCC = -0.84 with p-value = 2.23e-6, and PCC = -0.74 with p-value = 1.33e-4 in the control model (only using Hi-C data).

**Second***,* the active-tight and active-loose domains predicted by our modeling method are reproducible. Despite that active domains generally have lower pairing frequencies, we identify also in the new population some active domains with extremly high pairing frequencies. 61 active-tight domains are detected, 55 of those (90%) are detected as active-tight domains in the population with the original setting. 423 active-loose domains are detected in the new population, 417 of those (96%) are identical with the active-loose domains from the population with the original setting (**Fig. S12A**).

**Third***,* the conclusion of transcriptional efficiency for two subclasses of the active domains hold true (**Fig. S14A and B**) .

**Fourth**, also the conclusions about DNA replication timing are conserved **(Fig. S14C and D)**.

Note that, a structure population with an upper bound of only 3 times the radius (3X-model) failed to converge to a low violation score because many TADs are becoming too close and cause streric clashes. We did not try larger values like six times the diameter of the domain radius, because this large distance is equivalent to half of nuclear radius for many of TADs and not relevant for studying homolgue pairing.

## Robustness with respect to variation in the *fmax* parameter

The single parameter affecting the contact probability is the threshold value *fmax*, which defines the frequency at which a contact is formed at 100% in the structure population. Because we do not resolve chromatin at features smaller than a TAD domain we use the intra-TAD contact frequencies that constitute a stable TAD as a reference when defining *fmax* (section A.2 and A.3 from Supplementary materials). To demonstrate that our conclusions are valid when varying this parameter we increased the original *fmax* by a factor of 50% (section A.2 and A.3 from Supplementary materials), which essentially scaled down the input Hi-C probabilities *a_ij_* to 2/3 of the original values. We generated a population of 3,000 structures imposing all contacts *a_ij_* ≥ 0.04. All the main conclusions in the paper are well conserved as shown bellow.

1. The PCC for the average radial positions of euchromatin domains between the two populations is 0.96 (p-value < 2.2e-16) (the linear regression is shown in **Fig. S11C**). The PCC for the homolog domain distances between two populations is 0.93 (p-value < 2.2e-16, linear regression is shown in **Fig. S11D**).
2. Also the mean radial positions of each domain per chromosome are well conserved (**Fig. S12B**)
3. We repeated all the analysis regarding homologus pairing in the manuscript by using two different surface-to-surface distance cutoffs (cutoff_1≤ 150nm and cutoff_2 ≤ 170nm respecitively). These cutoffs reproduce a similar fraction of the average homoge distance than in the initial population (the average homolog distance decreases slightly in this new population of structures). The conclusions are all reproduced using both both these two cutoffs.

In conclusion, we can show that varying all relevant parameter settings does not affect our results. All results are highly reproducible under the variation of these parameter settings.

## References Supplementary methods

1. Sexton T, Yaffe E, Kenigsberg E, Bantignies F, Leblanc B, Hoichman M, Parrinello H, Tanay A, Cavalli G: **Three-dimensional folding and functional organization principles of the Drosophila genome.** *Cell* 2012, **148:**458-472.

2. Imakaev M, Fudenberg G, McCord RP, Naumova N, Goloborodko A, Lajoie BR, Dekker J, Mirny LA: **Iterative correction of Hi-C data reveals hallmarks of chromosome organization.** *Nat Methods* 2012, **9:**999-1003.

3. Langmead B, Salzberg SL: **Fast gapped-read alignment with Bowtie 2.** *Nat Methods* 2012, **9:**357-359.

4. Filion GJ, van Bemmel JG, Braunschweig U, Talhout W, Kind J, Ward LD, Brugman W, de Castro IJ, Kerkhoven RM, Bussemaker HJ, van Steensel B: **Systematic protein location mapping reveals five principal chromatin types in Drosophila cells.** *Cell* 2010, **143:**212-224.

5. Pickersgill H, Kalverda B, de Wit E, Talhout W, Fornerod M, van Steensel B: **Characterization of the Drosophila melanogaster genome at the nuclear lamina.** *Nat Genet* 2006, **38:**1005-1014.

6. Kinney NA, Sharakhov IV, Onufriev AV: **Investigation of the chromosome regions with significant affinity for the nuclear envelope in fruit fly--a model based approach.** *PLoS One* 2014, **9:**e91943.

7. Graveley BR, Brooks AN, Carlson JW, Duff MO, Landolin JM, Yang L, Artieri CG, van Baren MJ, Boley N, Booth BW, et al: **The developmental transcriptome of Drosophila melanogaster.** *Nature* 2011, **471:**473-479.

8. Tjong H, Li W, Kalhor R, Dai C, Hao S, Gong K, Zhou Y, Li H, Zhou XJ, Le Gros MA, et al: **Population-based 3D genome structure analysis reveals driving forces in spatial genome organization.** *Proc Natl Acad Sci U S A* 2016, **113:**E1663-1672.

**Supplemental Figures**

**
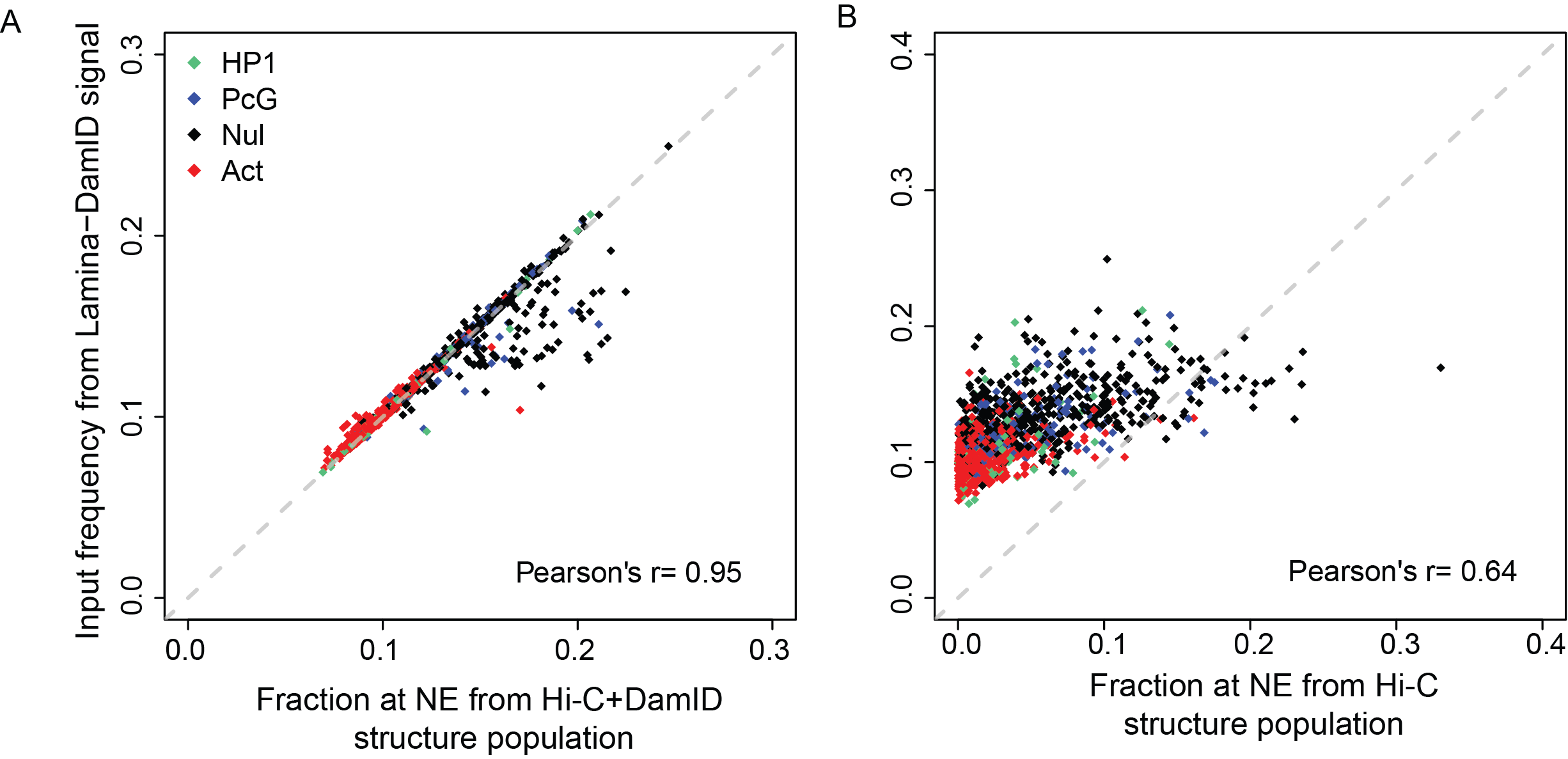
**

Figure S1. Agreement between the NE-association of euchromatin domains from lamina-DamID experiment and the models. (A) Fraction of domains at NE from population structure generated by data integration of Hi-C and lamina-DamID data well reproduces the input frequency derived from lamina-DamID data with Pearson’s correlation coefficient=0.95 and p-value< 2.2e-16. The points are colored according to the epigenetic classes. (B) Fraction of domains at NE from the control model with a structure population generated only from Hi-C data has a good correlation with the frequency derived from lamina-DamID data (Pearson’s correlation coefficient = 0.64 with p-value < 2.2e-16).


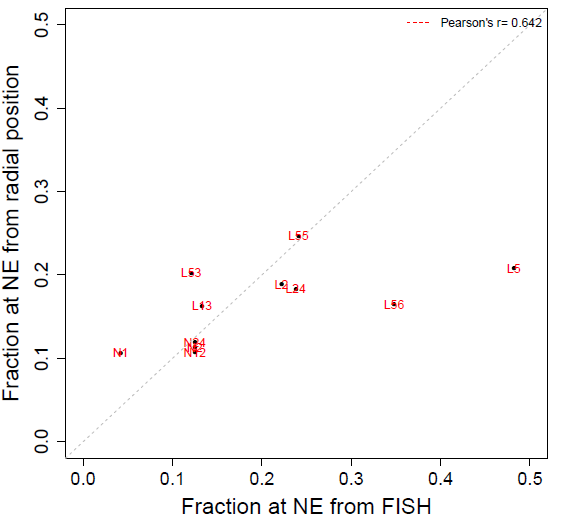

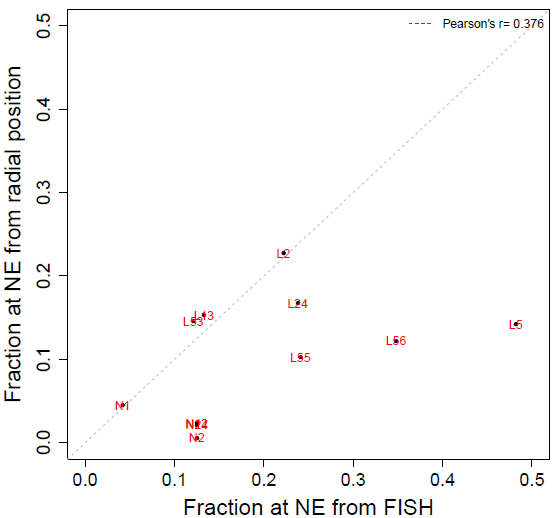


B

A

Figure S2. Agreement between the NE-association of individual loci from FISH experiment and the models. (A) Comparison of the NE association frequencies of individual loci from FISH experiment and from the model generated by data integration of Hi-C and lamina-DamID data. The NE association frequencies in the structure population agree well with FISH data for 11 loci (Spearman correlation coefficient=0.642 with p-value 0.03312). (B) Comparison of the NE association frequencies of individual loci from FISH experiment and the control model with a structure population generated only from Hi-C data. (Spearman correlation coefficient = 0.376 and p-value=0.2542).

A


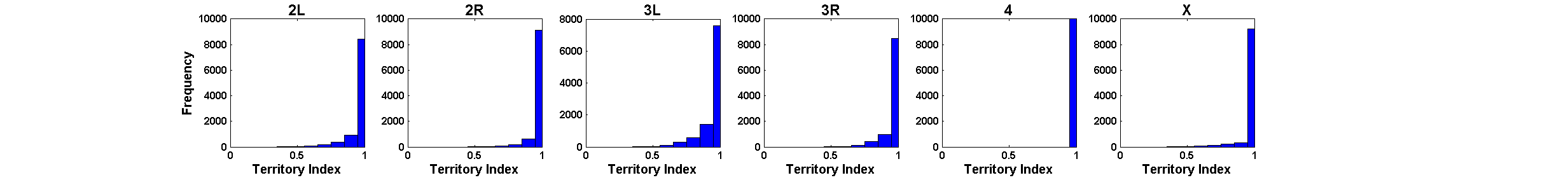


B


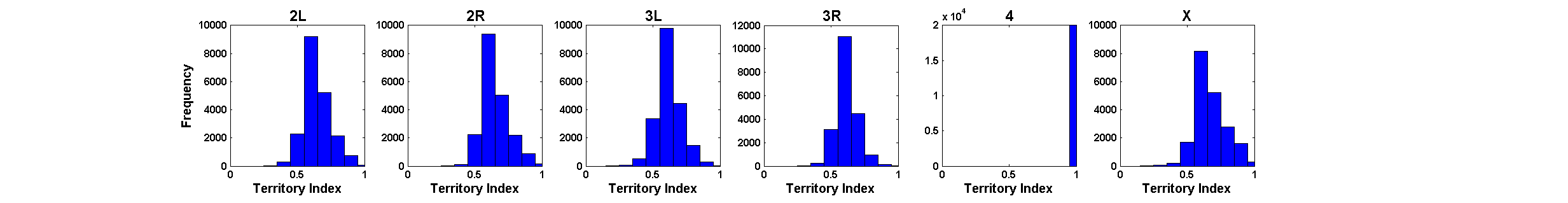


Figure S3. Territory index (TI). (A) TIs for the pairs of homologous chromosome arms. (B) TIs of each chromosome arm considering each homologues chromosome separately.


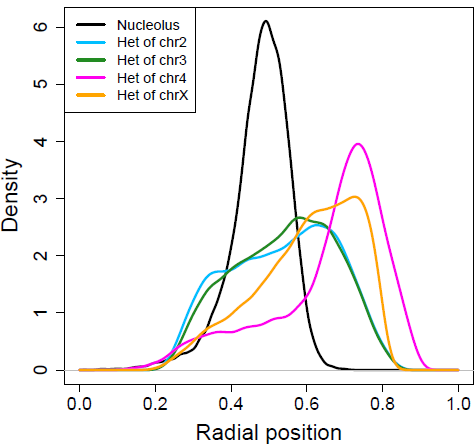

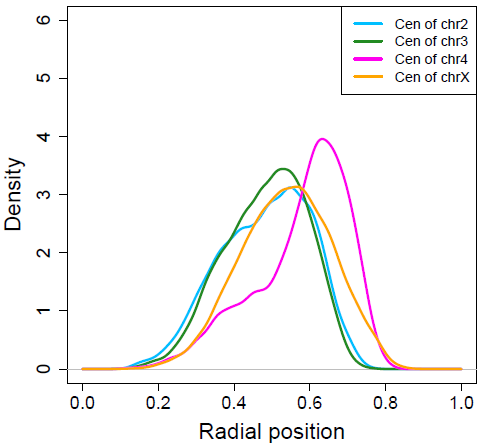


D

C

B

A


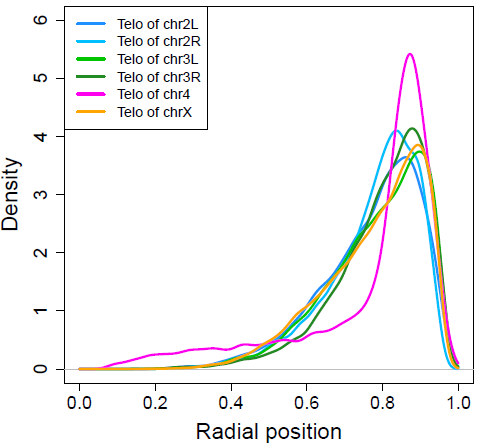

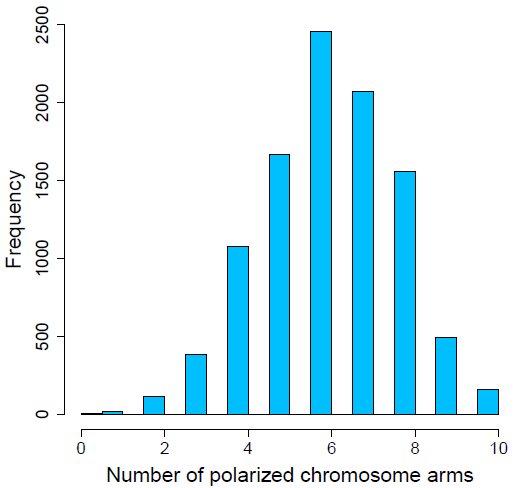


Figure S4. (A) Density plot of radial positions of the nucleolus and heterochromatin regions of different chromosomes. (B) Density plots of radial positions for centromeres. (C) Density plots of radial positions for peri-telomeric sequences. (D) Number of polarized chromosome arms (chr4 is excluded) among the population of structures.


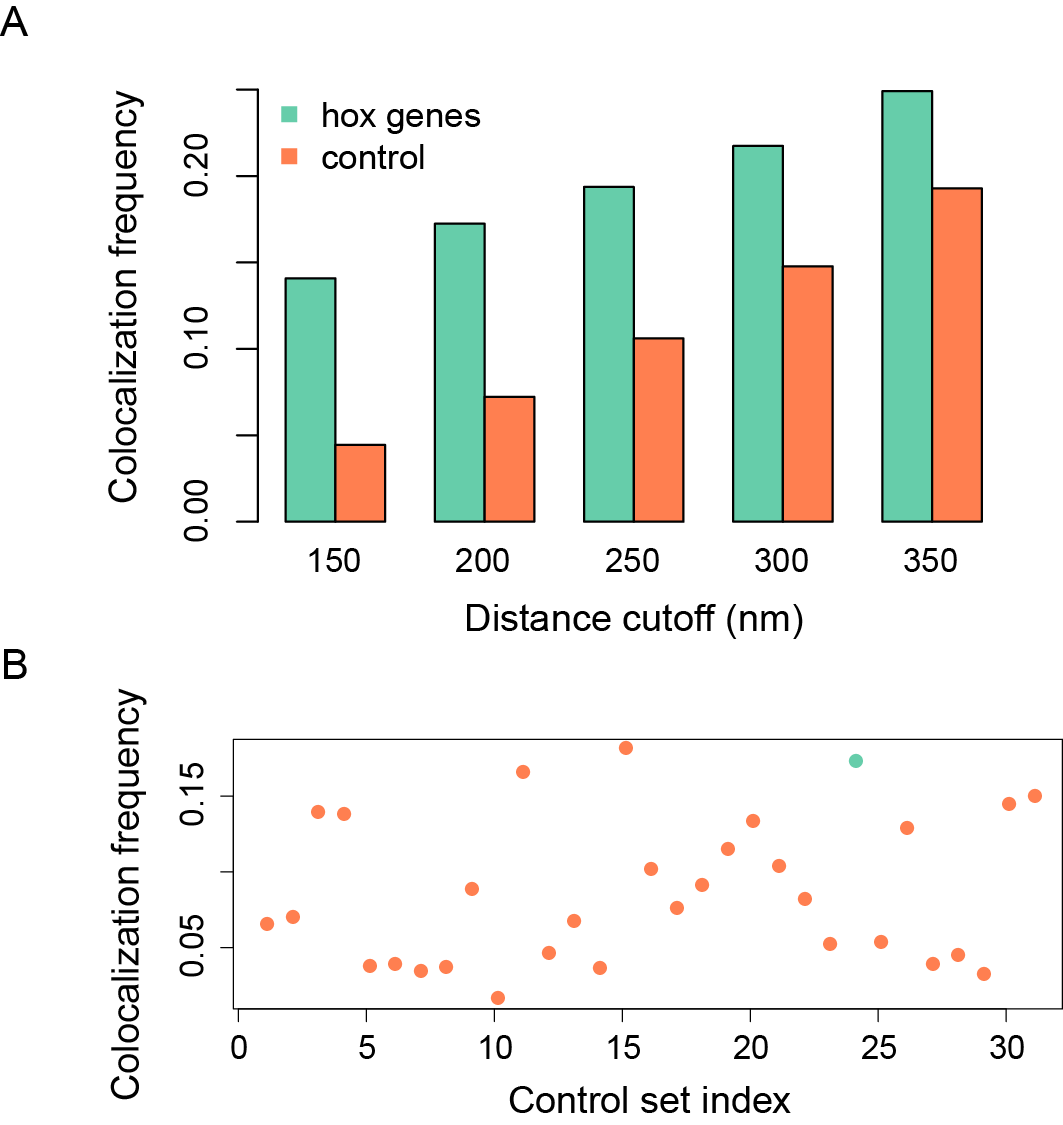


Figure S5. Hox gene clusters are prone to be co-localized comparing to control groups. (A) A hox gene cluster colocalization is defined if the closest surface-to-surface distance among any domain pairs of the two hox gene clusters is less than a distance cutoff. The hox genes are always colocalized in substantially higher percentage in our population than in the control groups across all distance cutoffs (from 150nm to 350nm, in steps of 50nm). (B) The hox gene clusters show contacts in higher percentage of structures in the population compared to all other control clusters except one (index 15) when 200nm is used as the cutoff.


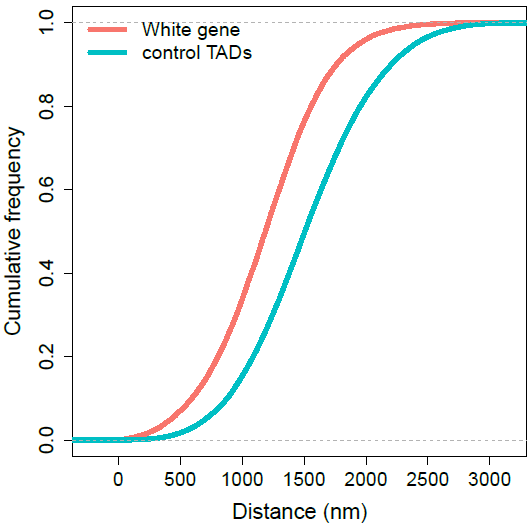

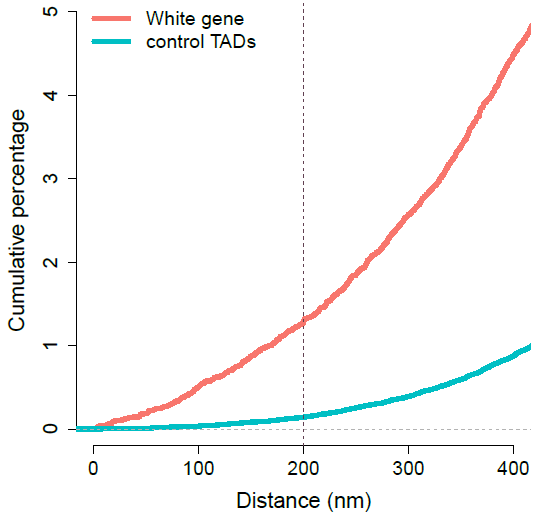


B

A

Figure S6. The *white* gene is prone to localize near to the pericentric heterochromatin. (A) Cumulative frequency plots for the distance of the *white* gene to its heterochromatin and of the control TADs to their corresponding heterochromatins. (B) Zoom the plot into the small distance, the *white* gene is 9-fold more frequently located proximal to pericentric heterochromatin (using 200 nm as a threshold), relative to control TADs.


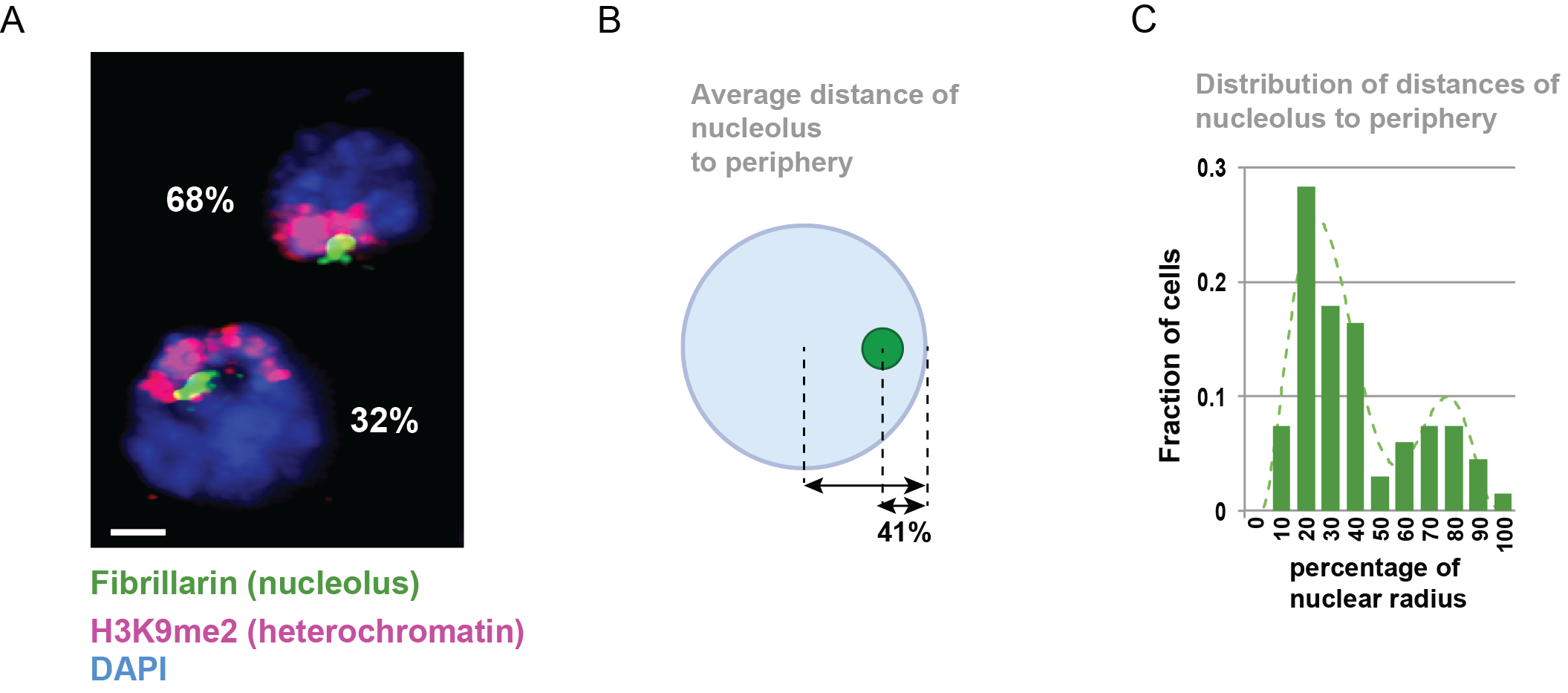


Figure S7. Nucleolus and heterochromatin positions in *Drosophila* Kc cells. (A) Immunofluorescence analysis with anti-Fibrillarin and anti-H3K9me2 antibodies, and DAPI staining for DNA (nuclear staining) shows the position and organization of the heterochromatin domain and the nucleolus in *Drosophila* Kc cells. The image shows a max intensity projection of two representative nuclei. Percentages indicate the population of cells in each configuration, *i.e.* with the nucleolus proximal to the nuclear periphery or more internal. N = 113 cells. Scale bar = 1 μm. (B) Quantification of the distance between the center of the nucleolus and the nuclear periphery shows the average position of the nucleolus relative to the center of the nucleus and the distribution of these distances in the cell population. N = 63 cells. (C) The distribution of the distance between the center of the nucleolus and the nuclear periphery show a bimodal distribution.


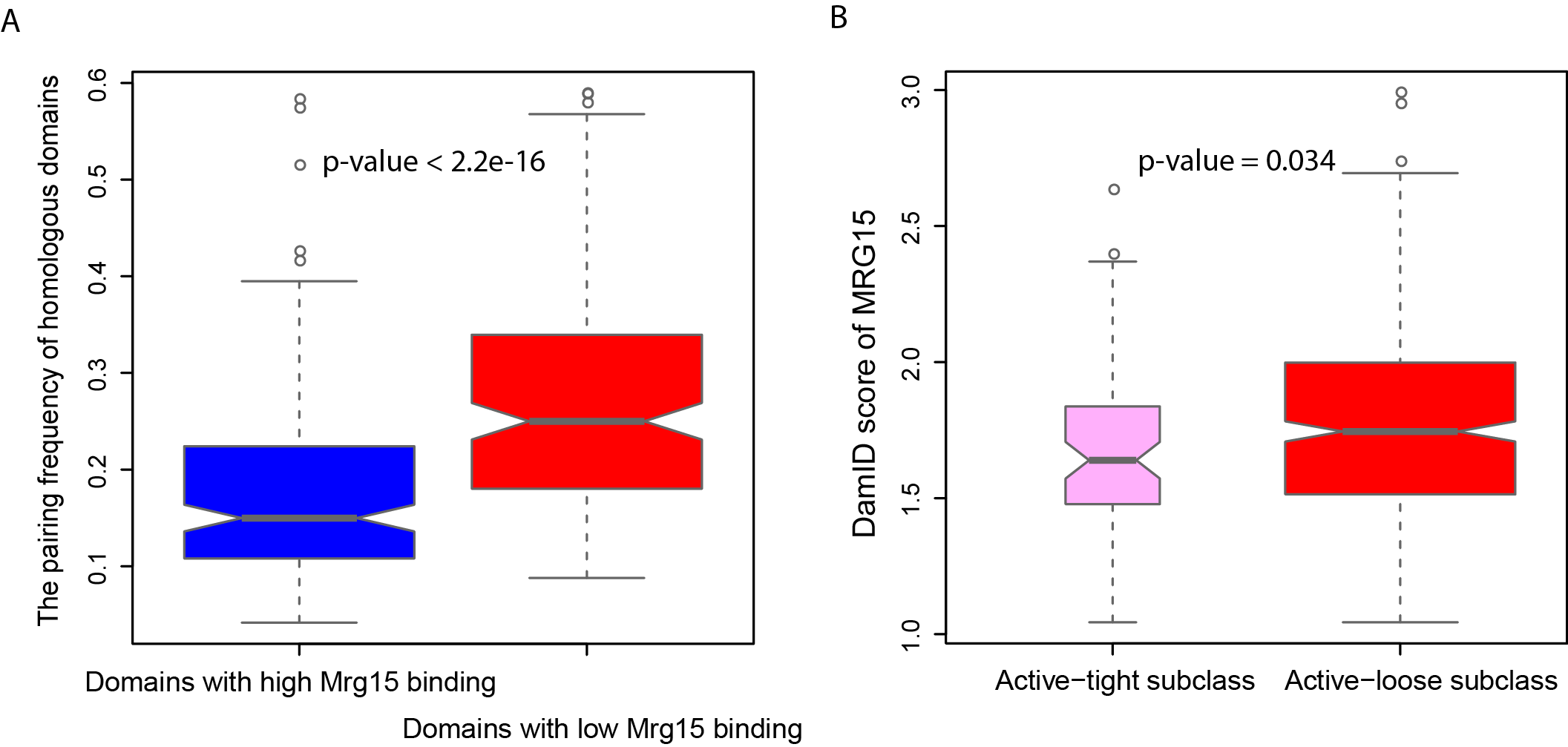


Figure S8. Inverse correlation between Mrg15 binding and homolog pairing frequency (A) Boxplot for the pairing frequency of domains with high and low Mrg15 binding. All domains are divided into 3 subsets based on their Mrg15 binding score. 293 domains are in the subset with high Mrg15 (top 25% binding scores); 293 domains are in the subset with low Mrg15 (bottom 25% binding score). The pairing frequencies for domains enriched with Mrg15 are significantly less than those for domains with low Mrg15 score (one-tailed Mann-Whitney U test, p-value < 2.2e-16). (B) Boxplot of Mrg15 score for the active-tight and active-loose subclasses. Active domains are divided into active-tight and active-loose based on their pairing frequencies (**Suppl. Methods C.6**). Active-tight domains, which have high pairing frequencies in our models, are significantly less enriched with Mrg15 comparing to active-loose ones (one-tailed Mann-Whitney U test, p-value = 0.034).


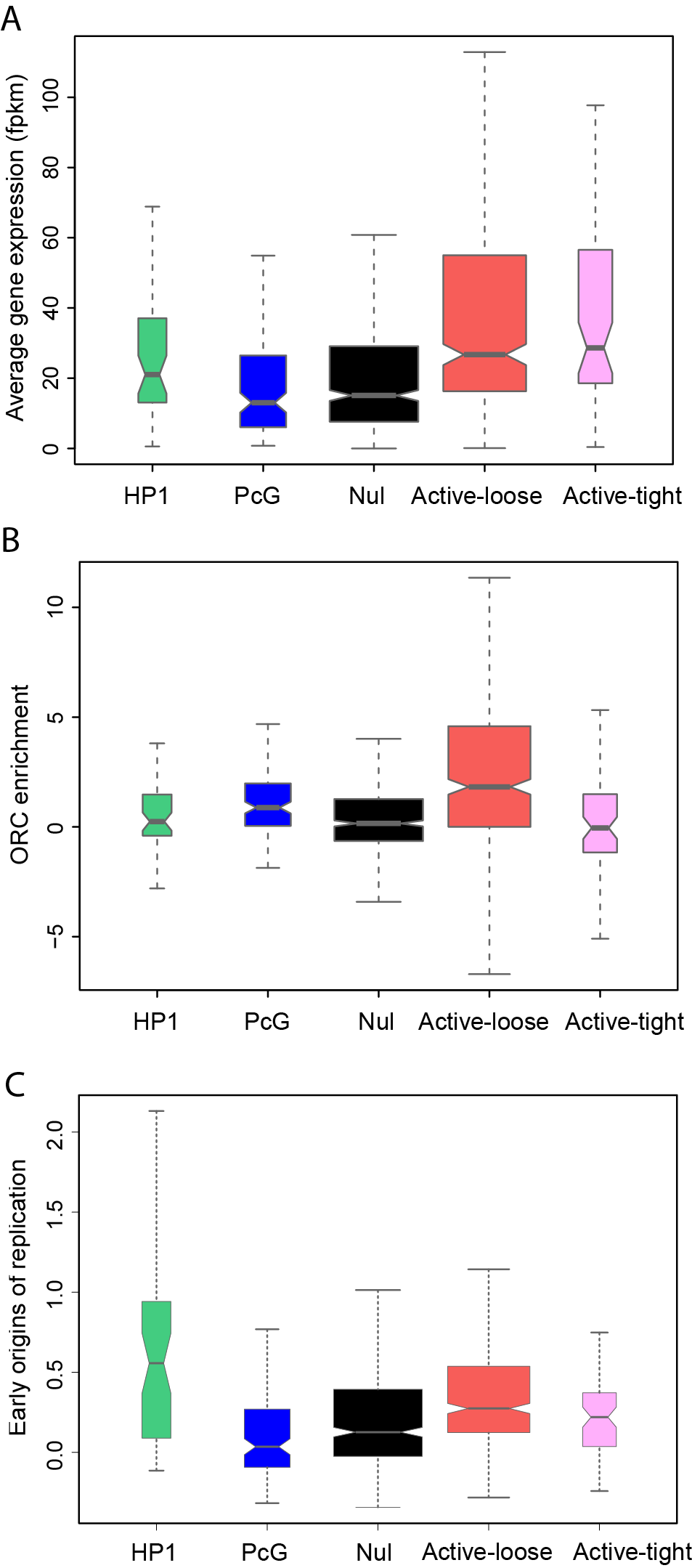


Figure S9. Transcriptional activity and DNA replication for all five classes (A) Domains in both two active subclasses have higher gene expression levels compared to those in the three repressive classes. (B) Domains in the active-loose subclass are more enriched with the replication complex ORC compared to the domains in the three repressive classes and the active-tight subclass. p-values: 1.48e-4, 7.59e-4, <2.2e-16 and 2.78e-5 respectively for HP1, PcG, Null and Active-tight. Active-tight subclass have no difference from NULL and HP1 classes with p-value = 0.36 and 0.26，but are even less enriched with ORC than the PcG class (p-value= 6.4e-4). (C) Active-loose domains are more enriched with early origins of replication comparing to PcG, Null and active-tight domains with p-value = 7.39e-15, 2.54e-13 and 2.92e-3 respectively, but less enriched with early origins of replication comparing to HP1 domains with p-value=7.1e-3. Compared to other classes, active-tight domains have no significant difference in replication timing from Null domains (p-value=0.13), but are replicated earlier than PcG (p-value=2.23e-4), and replicated later than HP1 domains (p-value=1.78e-4). Note, HP1 domains here do not belong to the pericentromeric heterochromatin.


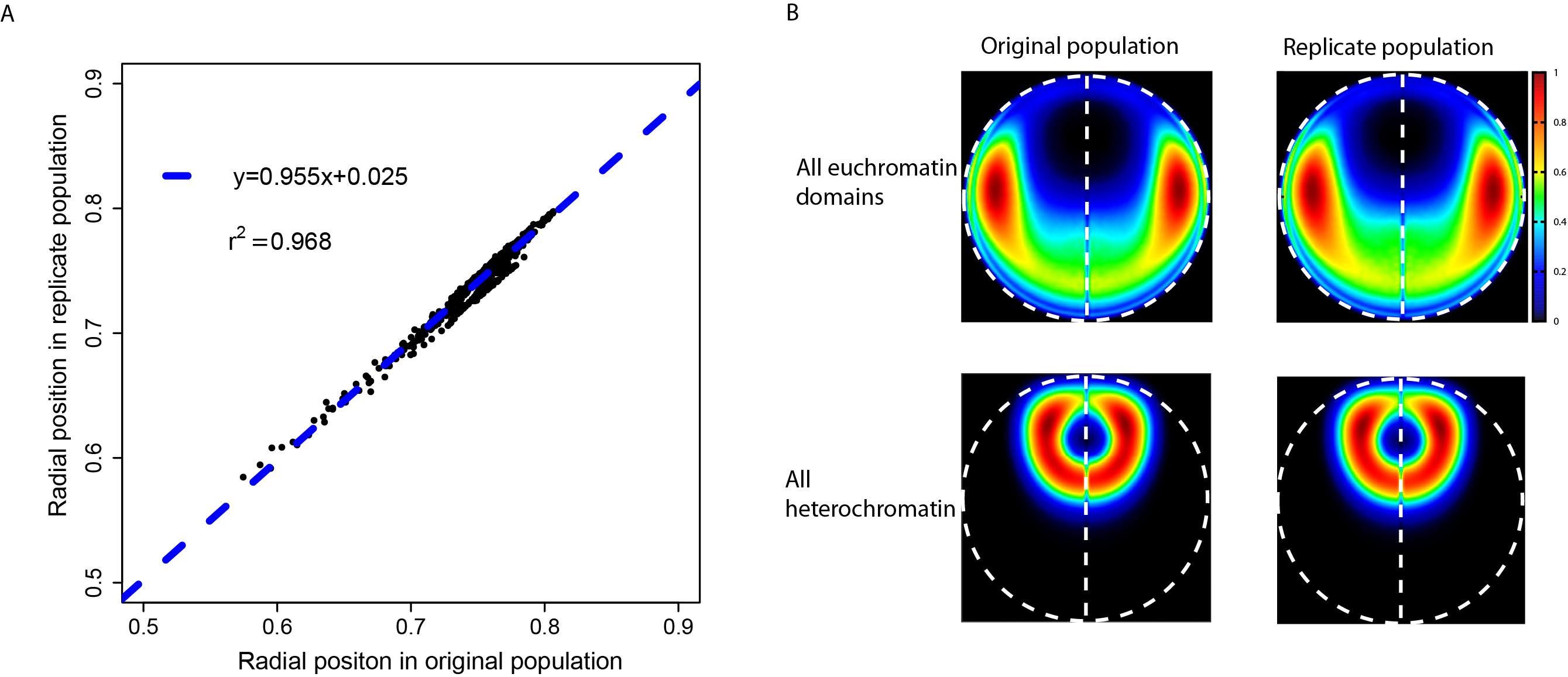


Figure S10. Reproducibility of the replicate simulations (A) Agreement of the average radial positions between two populations of structures. The Pearson’s correlation between them is 0.984, with p-value < 2.2e-16. (B) (Top panel) LPD plots of all euchromatin domains for the original population and the replicate population respectively. (Bottom panel) LPD plots of all heterochromatins for two populations of structures show highly consistent results.


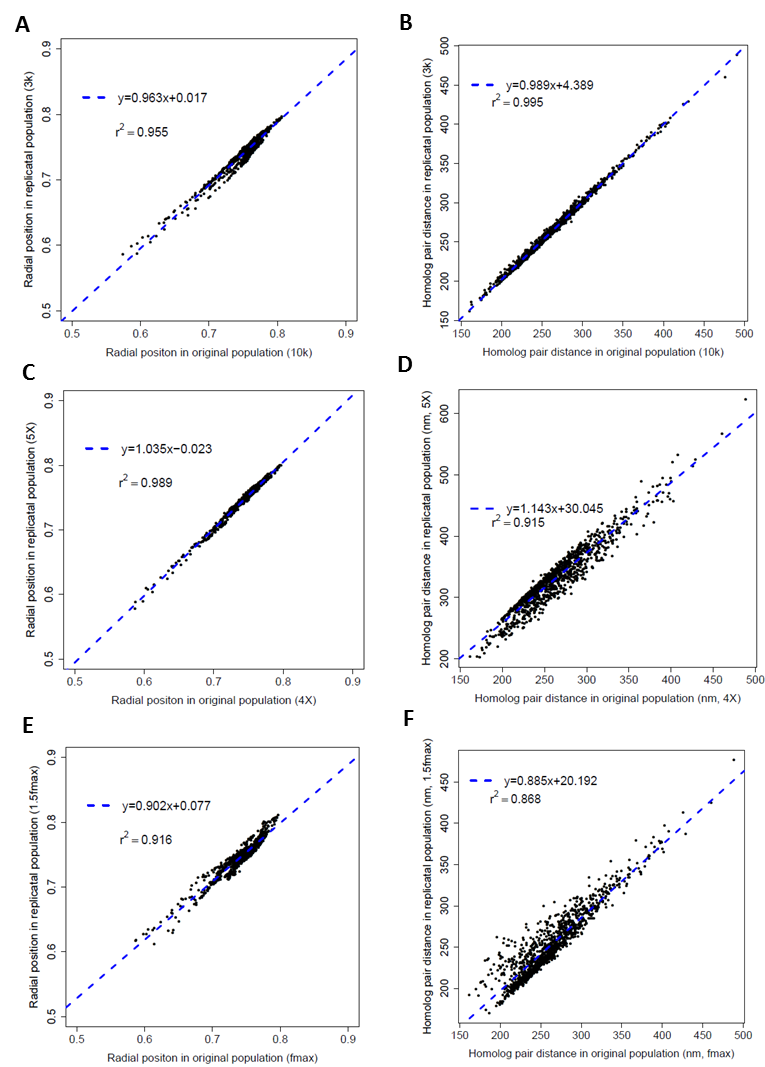


Figure S11. Reproducibility of the simulations with respect to population size, upper bound distance between homolog-pairs and variation in the *fmax* parameter (A, B) Comparison of average radial domain positions and average homologue pair distances between two independently calculated structure populations that vary in population size, namely with 10,000 structures (10k) and 3,000 structures (3k). (C, D) Comparison of average radial domain positions and average homologue pair distances between two independently calculated structure populations with different upper bound distance constraints between homolog-pairs, namely 4 times of the domain diameter (4x) and 5 times of the domain diameter (5x). (E, F) Comparison of average radial domain positions and average homologue pair distances between two independently calculated structure populations with different *fmax* parameter settings, namely the value obtained from assumption (*fmax*) and 50% increase from the original value (1.5*fmax*).


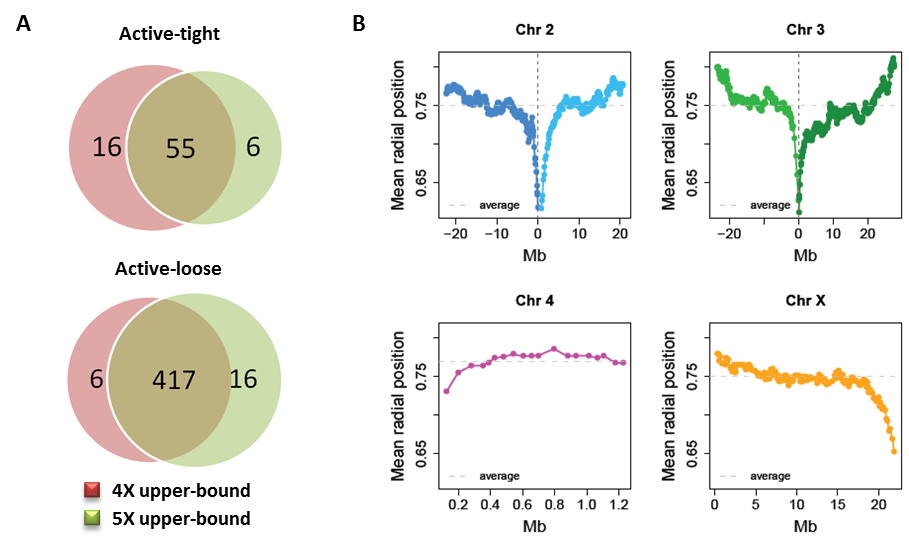


Figure S12. Robussness analysis of different parameter settings (A) The active-tight and active-loose domains are reproducible between the structure populations generated by varying the homolog-pair upper-bound distance constraint from 4 times to 5 times of radii diameter. 61 “active-tight” domains are detected in the new setting (5X-model), while 55 (90%) are overlapped with the ones from the population with original setting. 433 “active-loose” domains are detected in the new setting (5X-model), while 417 (96%) are overlapped with the ones from the population with original setting. (B) The chromosome shapes are well conserved in the population with 1.5*fmax* setting.


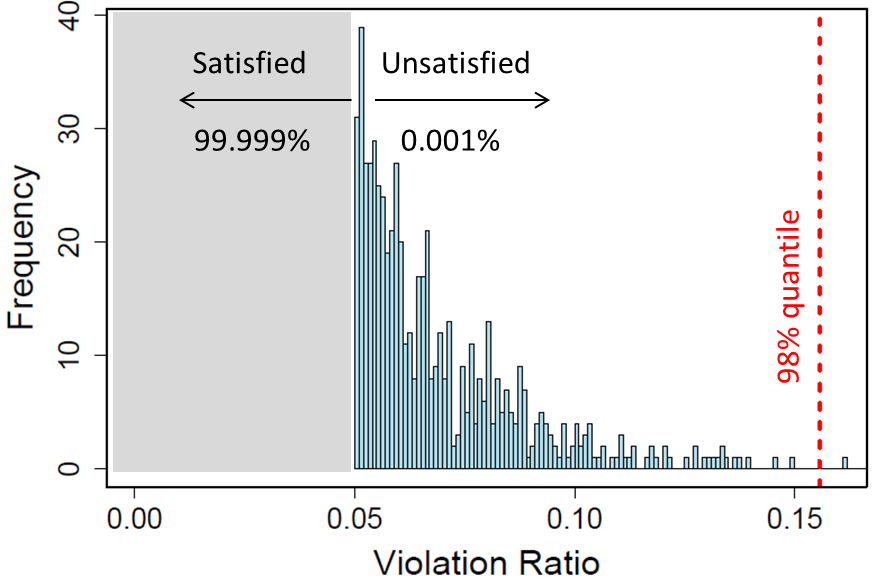


Figure S13. The violation analysis of constraints among the population of structures. There are 90,092,781 pairwise constraints in total (including the contact constraints from Hi-C and homolog-pairs), 14,529 (0.016%) among them are violated. We define a violation ratio for each constraint as (the center-to-center distance - the expected constraint distance (2 times of sum of radius)) divided by the expected constraint distance. We allow some tolerance, for example, to consider these constraints as satisfied if their violation ratios are less than 0.05, then 99.999% of the total constraints are satisfied. For 0.001% constraints not satisfied, the histogram of the violation ratio is plotted. The complete figure should have a long skew tail till the max ratio 1.05. The vertical line (98% quantile) shows that 98% of the violation ratios for unsatisfied constraints are smaller than 0.155. For lamina-NE constraints (not shown in the figure), there are 2,210,275 in total, among which 798 (0.036%) are violated with surface distance to NE larger than 50nm.


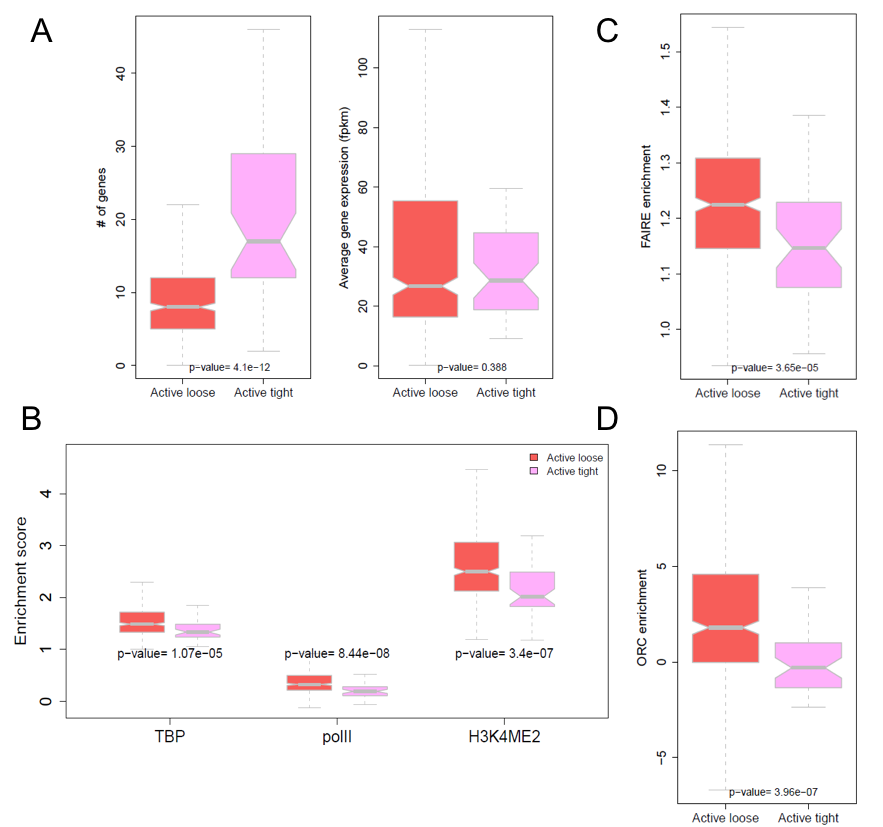


Figure S14. Transcriptional efficiency and DNA replication timing for genes in two sub-classes of the Active domains for 5X-model (Supp. Methods D.3). (A) (Left panel) Domains in the active-tight subclass contain significantly more genes than domains in the active-loose subclass. (Right panel) Genes in both sub-classes have similar average expression values. (B) TBP (TATA binding protein), PolII binding signal and H3K4me2 signals are more enriched in domains of the active-loose subclass. (C) FAIRE signal is significantly stronger in domains of the active-loose subclass. (D) ORC is significantly more enriched in domains of the active-loose subclass.
